# DNA methylation and lipid metabolism: an EWAS of 226 metabolic measures

**DOI:** 10.1186/s13148-020-00957-8

**Published:** 2021-01-07

**Authors:** Monica del C. Gomez-Alonso, Anja Kretschmer, Rory Wilson, Liliane Pfeiffer, Ville Karhunen, Ilkka Seppälä, Weihua Zhang, Kirstin Mittelstraß, Simone Wahl, Pamela R. Matias-Garcia, Holger Prokisch, Sacha Horn, Thomas Meitinger, Luis R. Serrano-Garcia, Sylvain Sebert, Olli Raitakari, Marie Loh, Wolfgang Rathmann, Martina Müller-Nurasyid, Christian Herder, Michael Roden, Mikko Hurme, Marjo-Riitta Jarvelin, Mika Ala-Korpela, Jaspal S. Kooner, Annette Peters, Terho Lehtimäki, John C. Chambers, Christian Gieger, Johannes Kettunen, Melanie Waldenberger

**Affiliations:** 1 Research Unit of Molecular Epidemiology, Institute of Epidemiology, Helmholtz Zentrum München German Research Center for Environmental Health, Ingolstaedter Landstraße 1, 85764 Neuherberg, Germany; 2 Institute of Epidemiology, Helmholtz Zentrum München German Research Center for Environmental Health, Neuherberg, Germany; 3 Department of Epidemiology and Biostatistics, Imperial College London, London, UK; 4 Department of Clinical Chemistry, Pirkanmaa Hospital District, Fimlab Laboratories, and Finnish Cardiovascular Research Center, Tampere, Faculty of Medicine and Health Technology, Tampere University, Tampere, Finland; 5 Department of Cardiology, Ealing Hospital, London North West University Healthcare NHS Trust, London, Middlesex UK; 6 Institute of Human Genetics, Helmholtz Zentrum München German Research Center for Environmental Health, Neuherberg, Germany; 7 Institute of Human Genetics, School of Medicine, Technical University Munich, Munich, Germany; 8 German Center for Cardiovascular Research (DZHK), Partner Site Munich Heart Alliance, Munich, Germany; 9 Chair of Microbiology, Technical University of Munich, Freising, Germany; 10 Center for Life Course Health Research, University of Oulu, Oulu University Hospital, Oulu, Finland; 11 Centre for Population Health Research, University of Turku, Turku University Hospital, Turku, Finland; 12 Research Centre of Applied and Preventive Cardiovascular Medicine, University of Turku, Turku, Finland; 13 Department of Clinical Physiology and Nuclear Medicine, University of Turku, Turku University Hospital, Turku, Finland; 14 Lee Kong Chian School of Medicine, Nanyang Technological University, Singapore, Singapore; 15 Institute for Biometrics and Epidemiology, German Diabetes Center, Leibniz Center for Diabetes Research at Heinrich Heine University Düsseldorf, Düsseldorf, Germany; 16 German Center for Diabetes Research (DZD), Munich-Neuherberg, Germany; 17 Chair of Genetic Epidemiology, IBE, Faculty of Medicine, LMU Munich, Munich, Germany; 18 Institute of Genetic Epidemiology, Helmholtz Zentrum München German Research Center for Environmental Health, Neuherberg, Germany; 19 Institute for Clinical Diabetology, German Diabetes Center, Leibniz Center for Diabetes Research at Heinrich Heine University Düsseldorf, Düsseldorf, Germany; 20 Division of Endocrinology and Diabetology, Medical Faculty, Heinrich Heine University Düsseldorf, Düsseldorf, Germany; 21 Department of Microbiology and Immunology, Faculty of Medicine and Health Technology, Tampere University, Tampere, Finland; 22 UKMRC-PHE Centre for Environment and Health, School of Public Health, Imperial College London, London, UK; 23 Department of Life Sciences, College of Health and Life Sciences, Brunel University London, London, UK; 24 NMR Metabolomics Laboratory, School of Pharmacy, University of Eastern Finland, Kuopio, Finland; 25 Computational Medicine, Faculty of Medicine, University of Oulu, Oulu, Finland; 26 Biocenter Oulu, University of Oulu, Oulu, Finland; 27 National Heart and Lung Institute, Imperial College London, London, UK; 28 Imperial College Healthcare NHS Trust, London, UK; 29 MRC-PHE Centre for Environment and Health, Imperial College London, London, UK; 30 Institute of Medical Biostatistics, Epidemiology and Informatics (IMBEI), University Medical Center, Johannes Gutenberg University, 55101 Mainz, Germany

**Keywords:** CpG site, VLDL, LDL, HDL, Lipoprotein sizes, Lipoprotein composition, Fatty acids, Myocardial infarction, Obesity, NMR

## Abstract

**Background:**

The discovery of robust and trans-ethnically replicated DNA methylation markers of metabolic phenotypes, has hinted at a potential role of epigenetic mechanisms in lipid metabolism. However, DNA methylation and the lipid compositions and lipid concentrations of lipoprotein sizes have been scarcely studied. Here, we present an epigenome-wide association study (EWAS) (*N* = 5414 total) of mostly lipid-related metabolic measures, including a fine profiling of lipoproteins. As lipoproteins are the main players in the different stages of lipid metabolism, examination of epigenetic markers of detailed lipoprotein features might improve the diagnosis, prognosis, and treatment of metabolic disturbances.

**Results:**

We conducted an EWAS of leukocyte DNA methylation and 226 metabolic measurements determined by nuclear magnetic resonance spectroscopy in the population-based KORA F4 study (*N* = 1662) and replicated the results in the LOLIPOP, NFBC1966, and YFS cohorts (*N* = 3752). Follow-up analyses in the discovery cohort included investigations into gene transcripts, metabolic-measure ratios for pathway analysis, and disease endpoints. We identified 161 associations (*p* value < 4.7 × 10^−10^), covering 16 CpG sites at 11 loci and 57 metabolic measures. Identified metabolic measures were primarily medium and small lipoproteins, and fatty acids. For apolipoprotein B-containing lipoproteins, the associations mainly involved triglyceride composition and concentrations of cholesterol esters, triglycerides, free cholesterol, and phospholipids. All associations for HDL lipoproteins involved triglyceride measures only. Associated metabolic measure ratios, proxies of enzymatic activity, highlight amino acid, glucose, and lipid pathways as being potentially epigenetically implicated. Five CpG sites in four genes were associated with differential expression of transcripts in blood or adipose tissue. CpG sites in *ABCG1* and *PHGDH* showed associations with metabolic measures, gene transcription, and metabolic measure ratios and were additionally linked to obesity or previous myocardial infarction, extending previously reported observations.

**Conclusion:**

Our study provides evidence of a link between DNA methylation and the lipid compositions and lipid concentrations of different lipoprotein size subclasses, thus offering in-depth insights into well-known associations of DNA methylation with total serum lipids. The results support detailed profiling of lipid metabolism to improve the molecular understanding of dyslipidemia and related disease mechanisms.

## Background

**Fig. 1:**
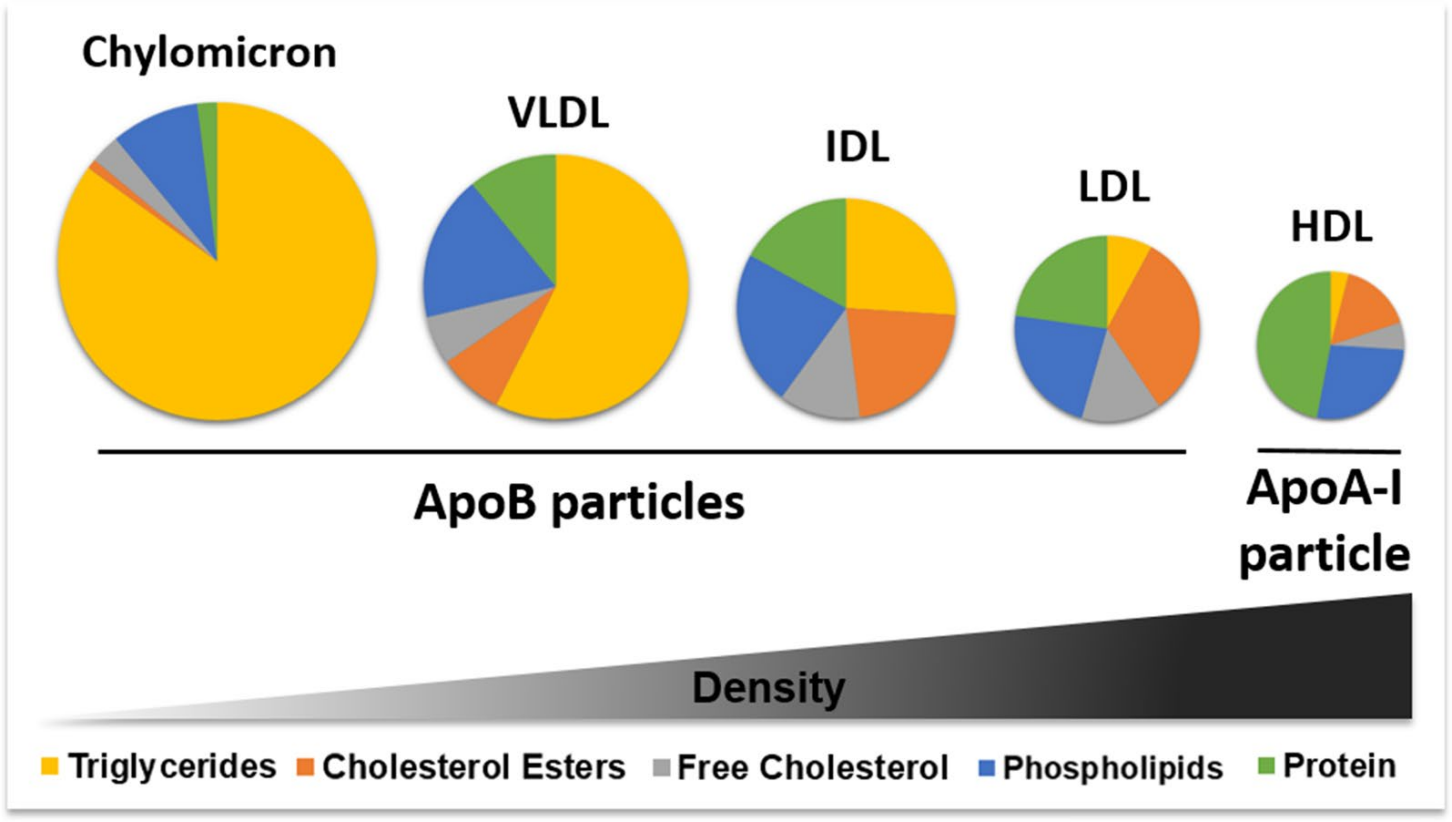
Lipoprotein composition. Lipoproteins can be categorized according to their size, composition, and density. The lattermost classifies them into chylomicrons, very low-density lipoproteins (VLDL), intermediate-density lipoproteins (IDL), low-density lipoproteins (LDL), or high-density lipoproteins (HDL). The less dense the particles are, the larger they are in size and the more lipids they contain [6–8]. Chylomicrons, VLDLs, IDLs, and LDLs are known as apolipoprotein B-containing particles, due to their main structural protein, while HDLs contain apolipoprotein A-I. Lipid and protein compositions are represented in approximated percentages [7]

**Fig. 2:**
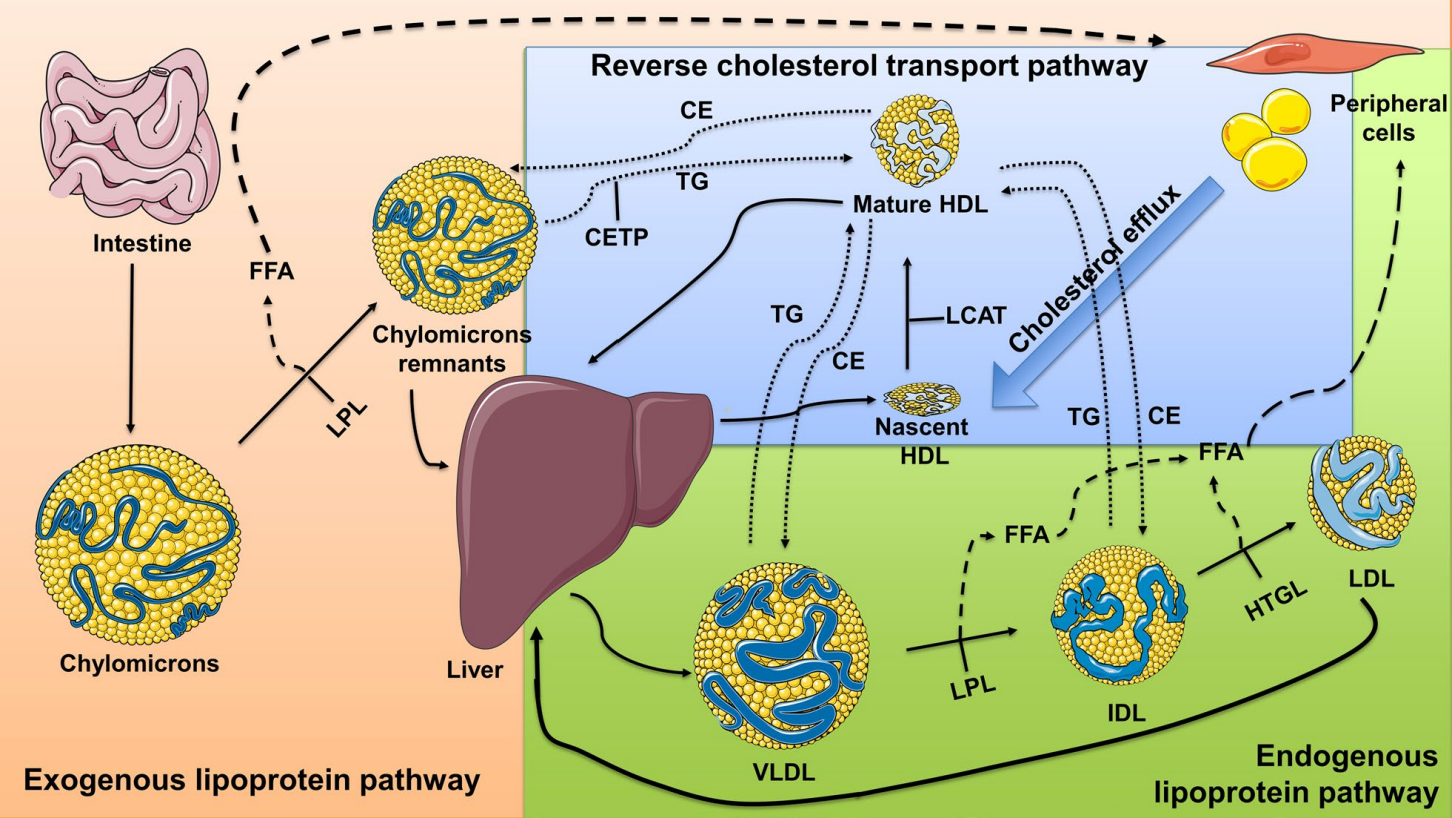
The roles of lipoproteins in the exogenous, endogenous, and reverse cholesterol pathways. Exogenous lipoprotein pathway (orange background): Lipid metabolism starts after food intake when chylomicrons are formed in the small intestine through the exogenous lipoprotein pathway [6]. Endogenous lipoprotein pathway (green background): The liver takes up chylomicron remnants, and the endogenous lipoprotein pathway then begins with the formation of VLDLs. VLDLs mainly transport TG from the liver to other tissues, in this process converting to IDLs and LDLs through the emission of fatty acids, and an increase in their cholesterol content [6]. Reverse cholesterol transport (RCT) pathway (blue background): The RCT delivers cholesterol from peripheral tissues back to the liver in both a direct and indirect manner. While in the direct RCT pathway effluxed cellular cholesterol is loaded onto HDLs for transportation, in the indirect RCT pathway cholesterol from HDLs is exchanged for TGs and transported by chylomicrons, VLDLs, and IDLs [6, 9]. In lipoprotein particles, yellow circles represent lipids and blue lines represent proteins. Solid arrows represent paths through which changes in lipoproteins occur. Segmented arrows represent paths that fatty acids follow. Dotted arrows represent paths that CE and TG follow. CE: cholesterol esters; TG: triglycerides; FFA: free fatty acids; CETP: cholesterol ester transfer protein; HTGL: hepatic triglyceride lipase; LCAT: lecithin:cholesterol acyltransferase; LPL: lipoprotein lipase

Dyslipidemia refers to abnormal levels of one or more lipids, such as plasma cholesterol, high-density lipoprotein cholesterol (HDL), low-density lipoprotein cholesterol (LDL), and/or plasma triglycerides (TG) in blood, leading to complex cardiometabolic diseases such as atherosclerosis, type 2 diabetes (T2D), or myocardial infarction (MI) [1–5]. Due to their poor solubility in blood, lipids are transported in lipoprotein particles that can be categorized according to their size, density, and composition as shown in Fig. 1 [6–8]. Lipoproteins are main players of the exogenous, endogenous, and reverse cholesterol transport pathways, thus contributing to lipid metabolism as illustrated in Fig. 2 [6, 9]. The smallest lipid molecules contained in lipoprotein particles are saturated and unsaturated fatty acids**.** Unsaturated fatty acids consist of monounsaturated fatty acids (MUFA) and polyunsaturated fatty acids (PUFA). Omega-3 PUFAs (e.g., docosahexaenoic acid (DHA)) have been linked to prevention of metabolic disorders, whereas for omega-6 PUFAs (e.g., 18:2 linoleic acid (LA)) inconsistent results exist [10]. Omega-3 to omega-6 FAs ratios and branched chain amino acids, such as isoleucine, are associated with metabolic outcomes [11–13].

There is mounting evidence that epigenetic mechanisms play an important role in the regulation of metabolic phenotypes and in other complex diseases [14–25], thus representing a possible therapeutic target [26–28]. While DNA methylation studies have highlighted several robustly replicated methylation markers of cardio-metabolic phenotypes [14–23], the full causal interplay is unknown. However, it has been proposed that most causal changes in methylation are a consequence rather than a cause of dyslipidemia and body mass index (BMI), therefore indicating that methylation may be more a biomarker of prevalent conditions rather than a predictor of incident conditions [20, 24, 25, 29–32].

To this point, most of the epigenome-wide association studies (EWAS) on metabolic measures of lipids have used conventional clinical measures, which reflect total concentrations of lipids in serum. However, the lipid composition, lipid concentration, and particle size of lipoproteins can be associated with disease risk independently of total lipid concentrations [33]. Therefore, EWAS on detailed lipoprotein measures are warranted. A helpful tool in this regard is nuclear magnetic resonance (NMR), which allows fine profiling of lipoproteins at a large scale [34–36]. NMR has successfully identified several new markers of metabolic disease [35, 36]. Up to now, three studies, including our previous investigation, have evaluated associations between serum metabolic measures and DNA methylation—with a limited sample size or a limited number of measures—identifying several loci linked to disease response mechanisms or environmental insults [29, 37, 38]. To identify robust associations between DNA methylation and metabolic measurements, we conducted an EWAS of 226 serum metabolic measures with subsequent replication in three independent cohorts (*N* = 5414 total). Metabolic measure-associated CpG sites were followed up for their associations with gene expression, relevant metabolic measure ratios, and disease endpoints in the discovery cohort only.

## Results

### Serum metabolic measures are associated with DNA methylation

**Fig. 3:**
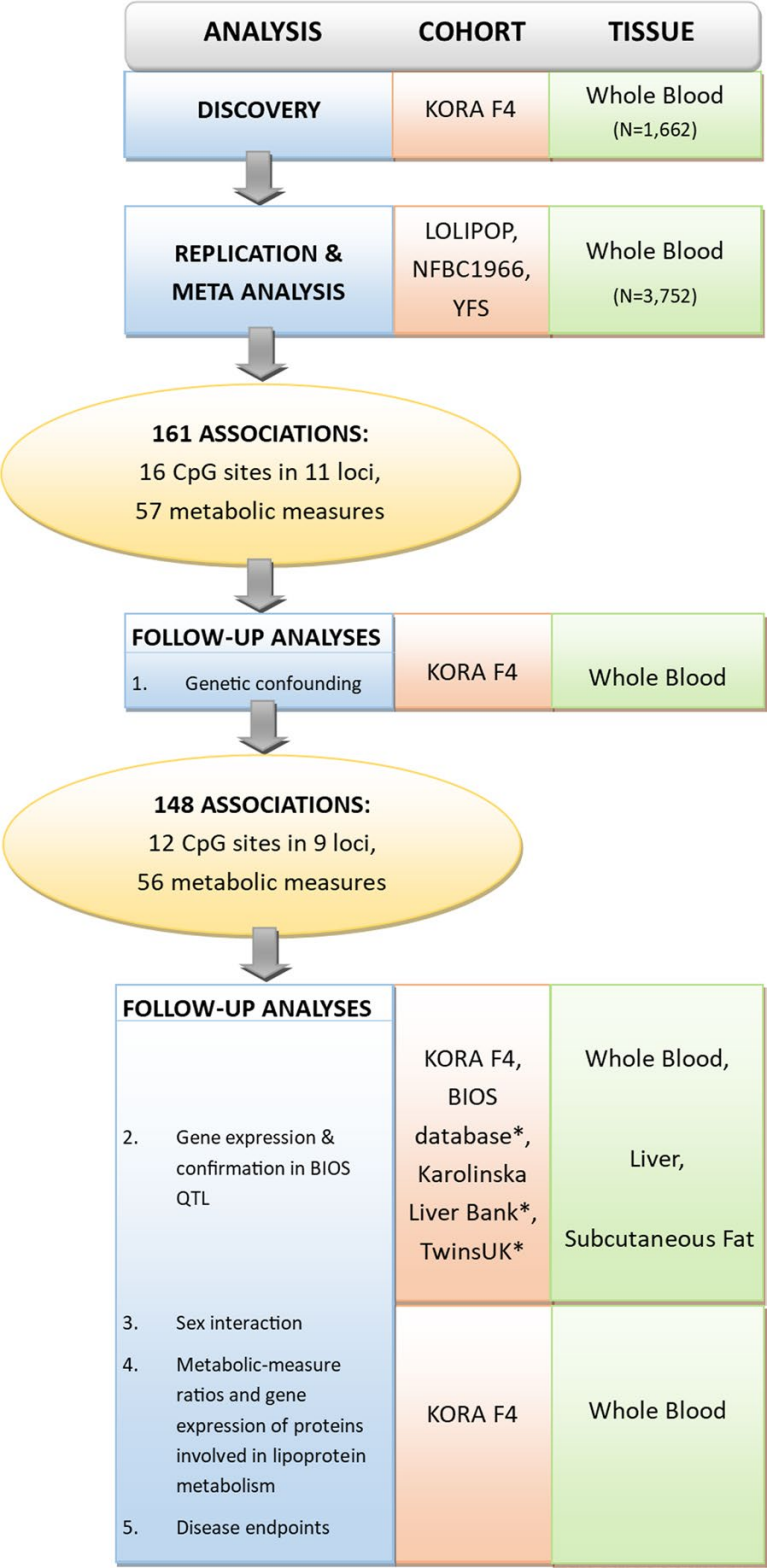
Study design. Displayed is the study design of the present work. It comprises a discovery and replication stage with subsequent follow-up analyses of the CpG sites associated with metabolic measures. All samples from the discovery and replication cohorts had methylation levels assessed using the 450 K BeadChip. *Data from publicly available databases

The main goal of our study was to identify robust associations between DNA methylation and metabolic measures, thus identifying the most promising CpG sites for follow-up investigations. Therefore, associations of metabolic measures with CpG methylation were first assessed in the discovery cohort (KORA F4) and subsequently examined in three independent cohorts (LOLIPOP, NFBC1966, and YFS) (Fig. 3). Characteristics of all cohorts are shown in Table 1. Mean ages ranged between 31.0 (NFBC1966) and 60.9 (KORA F4) years. At least in part due to their younger age at measurement, NFBC1966 and YFS participants were the healthiest, had no previous myocardial infarction, reported less hypertension, and less lipid-lowering drug intake.

**Table 1:** Characteristics of the study participants

	KORA F4 (*N* = 1662)	LOLIPOP (*N* = 2805)	YFS (*N* = 176)	NFBC1966 (*N* = 771)
Age (years)	61.0 (8.9)	51.4 (10.1)	44.2 (3.3)	31.0 (0.3)
Sex = male	49.0%	68.0%	37.5%	43.7%
BMI (kg/m^2^)^a^	28.1 (4.8)	27.7 (4.4)	25.9 (4.5)	24.4 (3.7)
Current smokers	14.4%	8.9%	16.5%	26.2%
Ex-smokers	41.5%	8.7%	25.6%	19.2%
Never smokers	44.1%	82.4%	58.0%	53.3%
Alcohol consumption (g/day)	15.5 (20.3)	6.38 (14.2)	0.716 (0.9)^b^	8.7 (14.3)
C-reactive protein (mg/l)	2.5 (5.2)	4.2 (7.2)	1.4 (2.4)	1.85 (3.3)
HDL-C (mmol/l)	1.7 (0.4)	1.3 (0.3)	1.7 (0.4)	1.7 (0.5)
LDL-C (mmol/l)	2.1 (0.6)	1.9 (0.6)	2.1 (0.6)	2.0 (0.7)
Total cholesterol (mmol/l)	5.7 (1.0)	4.9 (1.1)	5.3 (1.0)	5.5 (1.4)
Total TG^c^ (mmol/l)	1.6 (0.8)	1.5 (0.7)	1.2 (0.6)	1.2 (0.7)
VLDL-TG (mmol/l)	1.1 (0.8)	0.9 (0.6)	0.7 (0.6)	0.7 (0.5)
Total FA (mmol/l)	14.5 (2.4)	12.5 (3.0)	12.9 (2.3)	13.4 (3.5)
Hypertension^e^	45.5%	38.0%	19.3%	16.7%
Hospitalized myocardial infarction^e^	3.6%	3.9%	0.0%	0.0%
Intake of lipid-lowering drugs	16.2%	NA^d^	4.0%	0.0%

Displayed are characteristics of the discovery (KORA F4) and replication studies. Continuous and categorical characteristics are given as mean (standard deviation) for continuous variables and proportions for categorical variables. For every characteristic the *p* value for a difference between studies is < 0.001, given by a one-way ANOVA for continuous variables, and the Chi-square test for categorical variables

^a^BMI: body mass index

^b^Drinks per day (directly proportional to g/day)

^c^TG: triglycerides

^d^NA: variable not available

^e^Self-reported history

**Fig. 4:**
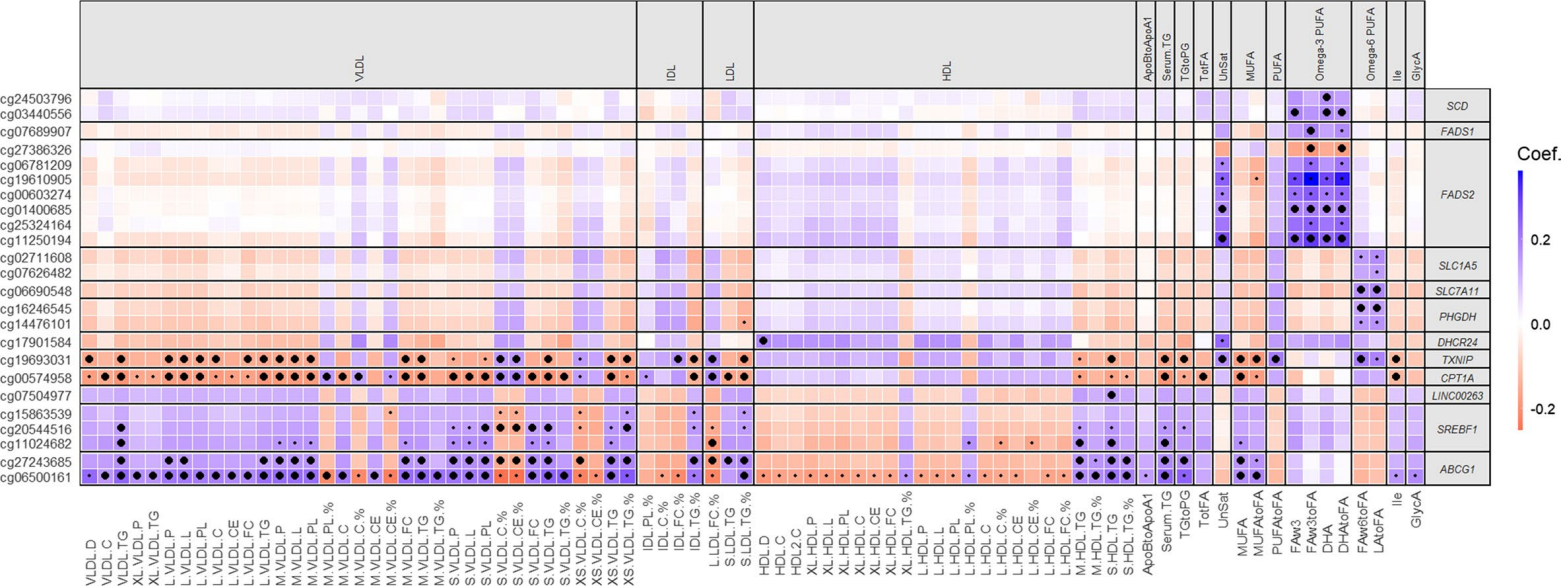
Associations between CpG sites and serum metabolic measures in the KORA F4 cohort and replication cohorts. Shown are all CpG sites and all metabolites with at least one significant association in the discovery analysis. Small dots: statistically significant association in the discovery analysis (KORA F4; *p* < 4.7 e−10). Large dots: significant associations in a meta-analysis of the results of the replication studies (LOLIPOP, NFBC1966, and YFS; *p* < 1.8e−4 and concordant direction of effect as discovery). Boxes are colored according to the signs and values of the coefficients after standardizing (subtraction of the mean, division by standard deviation) both the individual CpG site data and the log-transformed methylation data, to better visualize the relative strengths of association. D: diameter; C: cholesterol; CE: cholesterol esters; FC: free cholesterol; TG: triglycerides; P: particles; L: total lipids; PL: phospholipids; %: composition of a type of lipid to total lipids in a subclass of lipoprotein. See Additional file 1: Table S1 for a complete description of all abbreviations and the units of each serum metabolic measure

The discovery stage (*N* = 1662) consisted of 226 epigenome-wide association studies: 226 metabolic measures (Additional file 1: Table S1) versus methylation levels of 468151 CpG sites—a total of > 100 M possible associations. The discovery EWAS of metabolic measures revealed 282 significant associations, including 274 robust associations as defined by our sensitivity analyses (Fig. 4; Additional file 2: Table S2). These 274 associations had a percentage of explained metabolite-level variance ranging from 1.2% (cg19693031 in *TXNIP* with isoleucine) to 12.4% (cg19610905 in *FADS2* with omega-3 to FA ratio), covering 24 CpG sites annotated to 12 genomic locations (Fig. 4; Additional file 2: Table S2): cg06500161 and cg27243685 in *ABCG1* (*ATP-binding cassette sub-family G member 1*); cg11024682, cg15863539, and cg20544516 in *SREBF1* (*sterol regulatory element binding transcription factor 1*)*;* cg00574958 in *CPT1A* (*carnitine palmitoyltransferase 1A*)*;* cg19693031 in *TXNIP* (*thioredoxin interacting protein*)*;* cg17901584 in *DHCR24* [*24-dehydrocholesterol reductase*]; cg14476101 and cg16246545 in *PHGDH* (*D-3-phosphoglycerate dehydrogenase*)*;* cg07626482 and cg02711608 in *SLC1A5;* cg06690548 in *SLC7A11* (*solute carrier family 7 member 11*); cg07689907 in *FADS1* (*fatty acid desaturase 1*); cg00603274, cg06781209, cg11250194, cg19610905, cg25324164, cg01400685, cg27386326 in *FADS2* (*fatty acid desaturase 2*); cg03440556 and cg24503796 in *SCD* (*stearoyl-CoA desaturase*); and cg07504977 in the promoter region of *LINC00263,* a long non-coding RNA (lncRNA).

A meta-analysis (*N* = 3752) confirmed 161 of the 274 robust associations (58.8%), covering 16 of the 24 CpG sites found in the discovery step, annotated to 11 of the 12 genomic locations initially found, and 57 unique metabolic measures (Figs. 3 and 4; Additional file 2: Table S2). Across replication cohorts we observed consistent directions of effects, but effect sizes tended to be smaller in NFBC1966 and YFS (Additional file 3: Table S3; Additional file 4: Supplemental Results and Methods: Comparison across cohorts; Additional file 5: Table S4). For all 274 associations, the effect direction of the meta-analysis was concordant with the discovery direction, even those that were not successfully replicated, and 240 (87.6%) had a meta-analysis nominal *p* < 0.05. For eight CpG sites, corresponding to 4 genomic locations, associations did not replicate (cg15863539 in *SREBF1;* cg16246545 in *PHGDH;* cg07626482 and cg02711608 in *SLC1A5* (*Solute Carrier Family 1 Member 5*)*;* cg00603274, cg06781209, cg19610905, and cg01400685 in *FADS2*) (Fig. 4; Additional file 2: Table S2). No further CpG sites were associated with additional loci coding for enzymes or proteins directly involved in lipoprotein metabolism. Follow-up analyses involved only the 161 replicated associations, except for the correlation analyses of associated metabolic measures.

### Strong correlations between associated metabolic measures cluster DNA methylation in three groups

Strong correlations between associated metabolic measures found in the discovery cohort were observed for measures that showed the same directions of effect in associations with DNA methylation (Additional file 6: Figure S1). For instance, lipid compositions of larger VLDLs and TG measures in smaller HDLs showed strong positive correlations. We identified two groups of CpG sites showing most of their associations with lipoproteins (Fig. 4; Additional file 6: Figure S1). The first group consisted of CpG sites in *ABCG1*, *SREBF1*, and *LINC00263.* Methylation at these CpG sites showed most of their positive associations with lipid concentrations in ApoB lipoproteins, TG composition and concentration of HDLs, total serum TG, and MUFA measures. The second group consisted of CpG sites in *CPT1A* and *TXNIP,* showing most of their negative associations with lipid concentrations in ApoB lipoproteins, TG concentration of HDLs, total serum TG, MUFA measures, and isoleucine. A third group included CpG sites in *PHGDH*, *SLC7A11*, *FADS1/2*, and *SCD*. Methylation at these CpG sites only showed positive associations with PUFA measures and the degree of saturation of fatty acids.

Principal component analysis performed in the discovery cohort also points to these clusters of CpG sites. In EWAS of the top 8 principal components (PCs) of the metabolic measures data (see Additional File 4: Supplemental Results and Methods: Replication and meta-analysis; Additional file 7: Figure S2; Additional file 8: Figure S3), 6 CpG sites were found to be associated with PC1 (PC1 explaining 33% of the variance of the metabolite data) and 6 CpG sites were found to be associated with PC7 (2% explained variance) at a Bonferroni-corrected significance threshold of *p* = 0.05/(8 × 468151) = 1.34e−8 (Additional file 9: Table S5). No CpG sites were associated with both PCs. All sites were found in the discovery EWAS to be significant with at least one metabolite measure, and 9 of the 12 were found in the replicated results. The CpG sites associated with PC1 were annotated to the genes *TXNIP, SREBF1* (2 sites), *ABCG1* (2 sites), and *CPT1A*. The CpG sites associated with PC7 were mapped to *FADS2* (5 sites) and *SCD.*

### DNA methylation is associated with fatty acids and the lipid concentrations and compositions in lipoprotein subclasses

Of the 161 replicated associations, 159 were related to lipid metabolism and 121 involved lipoprotein subclasses, most of these being subclasses of ApoB lipoproteins (Fig. 4; Additional file 2: Table S2). When considering sizes of ApoB lipoproteins, extra-large VLDLs showed only two associations, while medium and small VLDLs were the most associated subclasses, with almost 60 associations altogether. All subclasses of ApoB lipoproteins, except extra-large and large VLDLs, had associations involving TG composition, with some subclasses additionally having associations with respect to cholesterol, cholesterol ester, and phospholipid composition. In terms of lipid concentrations of ApoB lipoproteins, almost all types of lipids showed associations in large, medium, and small VLDLs. In extra-small VLDLs and small LDLs, the concentration of TG was the only concentration associated with methylation. All associations for HDL lipoproteins, except for one featuring the diameter of HDLs, involved either TG composition or concentration of TG in medium and small HDLs. Associations with MUFAs and serum TG showed the same CpG sites and directions of effects as the associations pertaining to the ApoB lipoproteins. The CpG sites associated with PUFAs did not show associations with lipoproteins.

### CpG sites associated with metabolic measures have been linked to metabolic traits

To investigate common environmental and lifestyle-dependent drivers of the CpG site-metabolic measure associations, we performed searches in the EWAS Atlas and the MRC-IEU EWAS Catalog (Additional file 10: Table S6) [39, 40]. All CpG sites were found with at least one association in these databases, except for cg07689907 of *FADS1*. cg00574958 in *CPT1A* was found in the most unique publications (33 total), most associations cited being lipid-related and metabolic-related traits such as kidney disease and gamma-glutamyl transferase. The CpG sites of *SCD* have been less cited, appearing in only seven publications total, no outcomes being obviously related to fatty acids.

### Genetic effects on associations between DNA methylation and serum metabolic measures

We next performed follow-up analyses to test spuriousness caused by genetic confounding in the replicated associations, i.e., whether the replicated associations between DNA methylation and metabolic measures (at *p* = 4.7 e−10) are driven by genetic variants in *cis* of the CpG sites. Thirty of the 161 CpG-metabolite associations became non-significant due to the influence of *cis*-SNPs (*cis*-methQTLs) (Additional file 11: Table S7). For 17 of the 30 pairs, the results did not reflect strong evidence of genetic effects on the associations, as *p* values and coefficients changed only very little when adjusting for SNPs. However, for 13 of the 30 pairs (with CpG sites all located in the *FADS* cluster), the addition of a single SNP radically decreased the magnitude of the estimated effect size (and its *p* value) of the CpG site, likely indicating the association is being confounded by genetic effects (Additional file 11: Table S7). Because each association involving CpG sites within the *FADS* region showed strong evidence of being confounded by genetic factors, we eliminated these CpG sites from further analysis. This left a total of 12 associated CpG sites in 9 loci, 56 unique metabolic measures, and 148 total CpG-metabolic measure pairs carried forward to all further follow-up analyses (Fig. 3).

### DNA methylation associated with serum metabolic measures is linked to transcriptional differences in blood, adipose tissue, and liver

We then explored associations between CpG sites showing replicated associations, and gene transcripts within 1 Mb in blood, adipose tissue, and liver (Table 2; Additional file 12: Table S8).

**Table 2:** Results of gene expression analysis of CpG sites associated with metabolic measures

CpG site	CHR	Probe ID	CpG annotated gene	Transcript annotated gene	Distance	Beta value	*P*	*P* Bonferroni	*N*	Tissue
cg16246545	1	ILMN_1704537	*PHGDH*	*PHGDH*	0	− 2.77	1.44e−24	6.91e−22	634	Whole blood
cg24503796	10	ILMN_1689329	*SCD*	*SCD*	0	− 1.45	2.39e−08	1.15e−05	633	Whole blood
cg03440556	10	ILMN_1689329	*SCD*	*SCD*	0	− 0.57	7.43e−07	3.56e−4	609	Whole blood
cg06500161	21	ILMN_2329927	*ABCG1*	*ABCG1*	0	− 2.79	2.67e−07	1.28e−4	635	Whole blood
cg20544516	17	ILMN_1663035	*SREBF1*	*SREBF1*	1542	− 0.38	8.83e−10	4.59e−07	626	Subcutaneous fat
cg20544516	17	ILMN_1745806	*SREBF1*	*PEMT*	308200	0.27	1.64e−05	8.53e−3	626	Subcutaneous fat
cg20544516	17	ILMN_1811933	*SREBF1*	*SHMT1*	514197	− 0.37	2.85e−09	1.48e−6	626	Subcutaneous fat
cg20544516	17	ILMN_2328986	*SREBF1*	*SREBF1*	1208	− 0.28	1.26e−05	6.57e−3	626	Subcutaneous fat

Statistically significant associations between metabolic measure-associated CpG sites and expression of *cis-*transcripts in whole blood (KORA F4, Bonferroni-adjusted significance threshold *p* < 0.05/480 = 1.0e−4) and subcutaneous fat (TwinsUK study, *p* < 0.05/521 = 9.6e−5). “Probe ID” and “Transcript_annotated_gene” are the transcript ID and annotated gene from the Illumina annotation files; CHR: chromosome location of the CpG site; distance: distance between the CpG site and the transcript based on positions given in the annotation files; beta value: beta-coefficient; P: *p* value; P Bonferroni: Bonferroni-corrected *p* value; N: number of observations in the model. All coefficients are change in log2-transformed expression intensity per unit increase in methylation (beta value on 0–1 scale), except for subcutaneous fat, which is correlation assessed using the R-package rmcorr

In the discovery cohort, we investigated a total of 480 CpG site-transcript pairs in whole blood. cg06500161 in *ABCG1* and cg16246545 in *PHGDH* were negatively associated (Bonferroni *p* < 0.05/480 = 1.0e−4) with their corresponding gene transcript, results replicating those found in the BIOS QTL database [41] (Table 2; Additional file 12: Table S8). The association involving cg16246545 in *PHGDH* was slightly mitigated by adjustment for a *cis*-SNP, but remained strongly significant (*p* = 3.7e−14, results not shown). cg24503796 and cg03440556 in *SCD* were also negatively associated with *SCD* expression. Adjusting for SNPs in *cis* of the CpG sites had little or no effect on the results with regard to the expression results, except where noted.

We then performed an analysis using publicly available data for liver tissue (Karolinska Liver Bank cohort) for a total of 271 CpG site-expression probe pairs. In a limited sample of 92 individuals, no pairs were significant at a Bonferroni-corrected threshold (*p* < 0.05/271 = 1.8e−4), but three sites had negative associations with transcripts at FDR < 0.05: cg17901584 in *DHCR24* with a transcript of *TTC4*; and cg24503796 and cg03440556 in *SCD* with transcripts annotated to *CHUK* and *COX15*, respectively (Additional file 12: Table S8).

Again using publicly available data, we performed a similar analysis for subcutaneous fat (TwinsUK study) for a total of 521 pairs. Only cg20544516 in *SREBF1* showed significant associations at a Bonferroni-corrected threshold (*p* < 0.05/521 = 9.6e−5), with one transcript annotated to *PEMT*, one to *SHMT1,* and two annotated to *SREBF1.* The associations of cg20544516 with the two transcripts of *SREBF1* were replicated in the BIOS database. There were four additional significant associations at FDR < 0.05: cg24503796 in *SCD* with one transcript of *NDUFB8* and one of *PAX2*; cg20544516 in *SREBF1* with a transcript of *LOC201164*; and cg19693031 in *TXNIP* with a transcript of *DARS2* (Table 2; Additional file 12: Table S8).

### Limited to no evidence for sex specificity in DNA methylation-metabolic measure associations

In the discovery cohort, a CpG-by-sex interaction analysis of the replicated pairs revealed no Bonferroni-corrected significant (*p* < 0.05/148 = 3.4e−4) differences between men and women for the associations (Additional file 13: Table S9). However, nominally significant (*p* < 0.05) associations were found for 15 pairs, 14 of which involved small or very small VLDLs and CpG sites in either *SREBF1* or *CPT1A*.

### DNA methylation is linked to a variety of metabolic pathways

As a further approach to link enzymatic activity of selected metabolic pathways with CpG sites showing replicated associations, we examined 60 ratios of metabolic measures closely related to enzymatic substrates or products, or ratios linked to metabolic diseases in the discovery cohort (Additional file 4: Supplemental Results and Methods: Associations with metabolic ratios from additional pathways, Additional file 14: Table S10). A total of 189 associations were obtained for calculated ratios of metabolic measures (Bonferroni-corrected significance threshold of *p* < 0.05/(60 * 12) ≈ 6.9e−5). All 12 CpG sites were found to be associated with at least one ratio reflecting enzyme activity or linked to metabolic diseases, such as 25 associations relating to glucose metabolism and 38 involving branched-chain amino acids. However, most of the associations were obtained with lipid ratios and, among all assessed CpG sites, the lowest *p* values were largely for associations with ratios related to lipid metabolism. Therefore, we then tested associations of the 12 CpG sites with 30 transcripts of proteins directly involved in lipoprotein metabolism such as enzymes, transfer proteins, and lipid transporters, most of these located in *trans* to the CpG sites. Additional transcripts located within a ± 500 bp region were also included. We observed a total of four pairs showing associations (Bonferroni-corrected significance threshold of *p* < 0.05/(62 * 12) ≈ 6.7e−5) (Additional file 15: Table S11).

### DNA methylation associated with serum metabolic measures is linked to lipid-related clinical phenotypes

We further investigated the relevance of the replicated associations for lipid-related clinical phenotypes in the discovery cohort. Building on our previous publications [15, 25], we tested whether CpG sites associated with metabolic measures were related to type 2 diabetes (T2D), obesity, myocardial infarction (MI), or hypertension (Table 3; Additional file 16: Table S12). To explore the effects of lipid-lowering drugs on the relationships, we ran two models: one without this covariate (model 1) and one with (model 2). Statistical significance was based on a Bonferroni-corrected threshold of *p* < 0.05/(4 * 12) ≈ 1.0e−3.

**Table 3:** Results of the investigation into associations between CpG sites associated with metabolic measures and clinical outcomes, and the effect on associations when adjusting for lipid-lowering drug intake

CpG	Gene	Outcome	Model 1: Without adjustment for intake of lipid-lowering drugs				Model 2: Adjustment for intake of lipid-lowering drugs				Significant in
			Beta value M1	P_M1	Bonf P M1	OR (95% CI) M1	Beta value M2	P_M2	Bonf P M2	OR (95%_CI) M2	
cg19693031	*TXNIP*	Diabetes	− 0.56	4.33E−09	2.08E−07	0.57 (0.47–0.69)	− 0.59	1.32E−09	6.32E−08	0.55 (0.46–0.67)	M1 and M2
cg06500161^a^	*ABCG1*	MI	0.70	2.87E−05	1.38E−03	2.02 (1.45–2.8)	0.41	2.56E−02	1	1.5 (1.05–2.15)	M1 only
cg17901584	*DHCR24*	MI	− 0.65	4.37E−04	2.10E−02	0.52 (0.37–0.75)	− 0.17	4.04E−01	1	0.85 (0.57–1.25)	M1 only
cg06500161^b^	*ABCG1*	obesity	0.35	3.94E−08	1.89E−06	1.42 (1.25–1.61)	0.36	2.54E−08	1.22E−06	1.43 (1.26–1.63)	M1 and M2
cg27243685^b^	*ABCG1*	Obesity	0.29	5.56E−06	2.67E−04	1.34 (1.18–1.52)	0.30	3.96E−06	1.90E−04	1.35 (1.19–1.53)	M1 and M2
cg00574958^b^	*CPT1A*	Obesity	− 0.29	3.30E−06	1.58E−04	0.75 (0.66–0.85)	− 0.29	2.86E−06	1.37E−04	0.75 (0.66–0.84)	M1 and M2
cg07504977^b^	*LINC00263*	Obesity	0.21	1.02E−03	4.87E−02	1.23 (1.09–1.39)	0.21	1.04E−03	4.98E−02	1.23 (1.09–1.39)	M1 and M2
cg16246545^b^	*PHGDH*	Obesity	− 0.29	5.88E−07	2.82E−05	0.74 (0.66–0.84)	− 0.29	1.27E−06	6.11E−05	0.75 (0.67–0.84)	M1 and M2
cg06690548^b^	*SLC7A11*	Obesity	− 0.31	2.75E−07	1.32E−05	0.73 (0.65–0.83)	− 0.30	3.76E−07	1.80E−05	0.74 (0.66–0.83)	M1 and M2
cg11024682^b^	*SREBF1*	Obesity	0.34	1.26E−06	6.03E−05	1.4 (1.22–1.6)	0.34	1.34E−06	6.45E−05	1.4 (1.22–1.6)	M1 and M2

Associations of CpG site-clinical outcome in the KORA F4 cohort. Clinical outcomes: previous myocardial infarction, prevalent type 2 diabetes, obesity, and prevalent hypertension. Model 1: logistic regression model with clinical outcome as dependent variable and technically adjusted methylation value as independent variable, adjusted for the following covariates: age, sex, BMI (except for obesity model), C-reactive protein levels, hemoglobin A1c levels (except for diabetes model), history of myocardial infarction (except for the myocardial infarction model), smoking status, alcohol intake, current hypertension (except for the hypertension model), physical activity, white blood cell count, and estimated proportions of white blood cell types. Model 2: additionally adjusted for intake of lipid-lowering drugs (yes/no). Only statistically significant results are shown. P: *p* value; M1: model 1; M2: model 2; OR: odds ratio for a 1 standard deviation increase in methylation; CI: confidence interval

^a^Association using KORA F4 data in [15]

^b^Association using KORA F4 data in [25]

cg19693031 in *TXNIP* showed a strong association with T2D (*TXNIP*: odds ratio (OR) = 0.56 for a 1 standard deviation increase in methylation, 95% confidence interval (CI) = 0.47–0.69; model 1; Table 3), whereas seven CpG sites across six loci were significantly associated with obesity (smallest *p* value = 2.8e−7 for a site found in *SLC7A11*), among them one CpG site in *PHGDH*. The associations with both T2D and obesity were largely independent of lipid-lowering drug intake.

Associations with MI tended to be partially or completely mitigated when adjusting for the intake of lipid-lowering drugs. cg06500161 in *ABCG1* was positively associated with MI (*p* = 2.9e−5, OR = 2.02, CI = 1.45–2.80, model 1), but the association lost significance when adjusting for lipid-lowering drugs, with a large reduction in the odds ratio (*p* = 0.025, OR = 1.50; CI = 1.05–2.15). Similar effects were observed for cg17901584 in *DHCR24* (*p* = 4.4e−4 for model 1, effect disappearing completely in model 2 with *p* = 0.4).

No CpG sites were associated with hypertension. Adjusting for SNPs in *cis* of the CpG sites had little or no effect on the results with regard to clinical phenotypes**.**

We additionally generated receiver operating characteristic (ROC) curves for each of the CpG sites-clinical phenotype pairs significantly associated in model 1 or model 2 (Table 3; Additional file 17: Table S13; Additional file 18: Figure S4). For each pair and each model (M1 or M2), we plotted the ROC curve and calculated the area under the curve (AUC) for the model without the CpG site and for the model with the CpG site, to see whether the addition of the CpG site to the model significantly increases the model’s ability to predict the outcome. After incorporating the associated CpG sites into the models, five CpG site-outcome pairs for M1 and four for M2 showed nominally significant (*p* < 0.05) increases in AUC, e.g., cg19693031 in *TXNIP* showed an AUC = 0.813 without CpG site, and AUC = 0.834 with CpG site (*p* = 0.011).

## Discussion

This EWAS of 226 mostly lipid-related serum metabolic measures is the largest to date incorporating the different lipid concentrations and lipid compositions of lipoprotein subclasses. Our EWAS revealed 161 replicated associations between 16 CpG sites in eleven loci and 57 unique metabolic measures. All of the eleven epigenetic loci have been previously found to be associated with metabolic traits and processes, primarily based on clinical and biochemical measurements of composite blood lipids [14–20, 22–25, 29, 37, 38, 42, 43]. Here, we uncover novel findings with regard to specific features of lipoprotein subclasses such as the lipid compositions and concentrations of each type of lipid in lipoproteins, giving deeper insights into the underlying biology of previous associations.

Results related to ApoB lipoproteins, particles involved in the endogenous lipoprotein pathway, suggest that DNA methylation is intertwined with the changes in lipid compositions and concentrations that all sizes of VLDLs, IDLs, and LDLs undergo along the pathway. Methylation at CpG sites in *ABCG1*, *SREBF1*, *CPT1A,* and *TXNIP* had most of their associations with ApoB lipoproteins. Although methylation at CpG sites of those genes showed additional associations with HDLs, MUFAs, or isoleucine, their primary relationships seem to be with ApoB lipoproteins. *CPT1A* was the only gene whose CpG site showed associations with ApoB lipoproteins but not with HDLs. Methylation at cg00574958 in *CPT1A* was negatively associated with the concentration of almost all types of lipids and the TG composition in VLDLs, IDLs, and LDLs, adding evidence of a possible link between hypermethylation at this site and healthier metabolic phenotypes [44, 45]. Methylation at cg00574958 was also negatively associated with the TG composition of small LDLs. As smaller LDLs easily diffuse through the arterial wall, their low-TG load promotes increased cholesterol uptake and therefore impedes atherosclerosis development [46–48]. *CPT1A* is highly expressed in the liver, where it initiates mitochondrial oxidation of long-chain fatty acids and therefore contributes to lower serum TG levels, suggesting a relation between a higher *CPT1A* expression and lower serum TG levels [49]. Although no association between methylation at cg00574958 and expression of *CPT1A* was observed, this CpG site was negatively associated with serum TG and MUFA levels, the lower levels of which in turn limit TG acquisition by lipoproteins. Furthermore, albeit only nominally significant, sex-varying associations of methylation at cg00574958 with small and very small VLDLs could partially explain reported lower concentration and average size of circulating VLDLs in women compared to men [50]. In line with prior observations of sex-specific effects for *Cpt1a* in rodent lipid metabolism [51, 52], our results suggest a link between hypermethylation at cg00574958 and anti-atherogenic traits, possibly emphasized in females, further supporting that hypermethylation at this CpG site might be linked to healthier metabolic outcomes.

The reverse cholesterol transport (RCT) pathway delivers cellular cholesterol back to the liver in both a direct and indirect manner. The players of the direct RCT are HDLs, which are loaded with effluxed cholesterol from cells [9]. Apart from one association of larger HDL diameters with methylation at a CpG site in *DHCR24,* all other HDL associations found involved the TG concentrations and compositions in small and medium HDLs, and CpG sites in *ABCG1, SREBF1, LINC00263*, and *TXNIP*. HDLs that are TG-rich promote the clearance of circulating HDLs [46, 53, 54]. Therefore our findings might be related to the impairment of the RCT through larger HDLs and to associations of CpG sites in *ABCG1, SREBF1,* and *TXNIP* with adiposity-related traits [20, 24, 44, 45, 55]. *ABCG1, SREBF1*, and *TXNIP* were additionally associated with ApoB lipoprotein features and fatty acids. While these three genes have recently caught a lot of attention as they have been frequently found to be associated with metabolic traits [15–18, 20, 22–25, 29, 37, 44, 55], only sparse attention has been paid to a potential role of *LINC00263* in metabolic features [25, 32, 43]. In this study, *LINC00263* was the only lncRNA associated with metabolic measures. Notably, methylation at cg07504977 in *LINC00263* was solely associated with the TG concentration in small HDLs and no other metabolic measures, suggesting a specific role underlying previous general associations. First studies propose that *LINC00263* is a sex-specific oncogene [56]. Its involvement in metabolic disturbances is plausible as other genes involved in both cancer and metabolic disturbances have been reported [57]. No associations between methylation at cg07504977 and expression of *LINC00263* or other transcripts have been identified by us or by others, perhaps because of its absence on most commercial arrays. *LINC00263* interacts with at least 100 mRNAs, lncRNAs, miRNAs, and transcription factors [56], a common characteristic of lncRNAs [58]. The location of cg07504977 overlaps an active histone mark region within the promoter of *LINC00263* [43], suggesting a role of methylation at this CpG site in the lncRNA transcription. Thus, it seems feasible that methylation at cg07504977 could affect *LINC00263* expression, which in turn could directly adsorb or regulate the expression of miRNAs, and indirectly regulate the expression of miRNA target genes, e.g., miR-128, which directly inactivates *ABCG1* [59, 60]. Our results link methylation at cg07504977 to obesity and support this CpG as an emerging target for metabolic outcomes.

The indirect RCT pathway exchanges TG and cholesterol esters between VLDLs, LDLs, and HDLs, and is promoted by high levels of VLDLs with subsequent HDL clearance [6, 9]. Our results highlight methylation at cg06500161 in *ABCG1* as a biomarker entangled not only in the endogenous lipoprotein and the direct RCT pathways, but also in the indirect RCT pathway. cg06500161 showed the highest number of associations involving ApoB lipoproteins and HDLs and the lowest *p* values among all assessed CpG sites. In this study, cg06500161 was one of two CpG sites that exhibited associations with their respective gene transcript, metabolic measure ratios, and disease endpoints. Moreover, transcripts of *ABCG1* were additionally associated with CpG sites located in *trans* and annotated to *DHCR24* and *CPT1A. *Novel results for methylation at cg06500161 include associations with the lipid concentrations of all types of lipids and the TG compositions in ApoB lipoproteins, and associations with the TG composition and TG concentration in smaller HDLs. Additionally, in line with the known function of ABCG1 in controlling the bioavailability and activity of the TG-hydrolysis enzyme lipoprotein lipase [61], the analysis of metabolic measure ratios showed the lowest *p* values for ratios involving serum total TG levels. CpG methylation has shown to be driven by TG levels and not vice versa [24]. Although ABCG1 promotes the net cholesterol efflux to larger HDLs in the direct RCT pathway [62, 63], and associations with *ABCG1* transcription were found, no associations of cg06500161 with the cholesterol concentration or composition in HDLs were observed. Therefore, methylation at cg06500161 might be linked to a lower activity of TG-hydrolysis enzymes (e.g., lipoprotein lipase), which in turn enhances the indirect RCT pathway, and impedes observation of associations with cholesterol features in HDLs [15, 25]. However, a precise role of cg06500161 in lipid metabolism remains to be elucidated, as does that of lipid-lowering drugs in the relationship. The previously hypothesized influence of statins on methylation at this site [64], and its mediation on the association between statins and type 2 diabetes [65], suggest that methylation at cg06500161 could lie on the causal path between the apparent mitigating effect of lipid-lowering drugs and its association with MI.

The chemical structure of fatty acids (FA) allows their categorization according to their saturation into MUFAs or PUFAs. In blood, FAs are transported by ApoB lipoproteins and HDLs. Although not fully understood, it has been proposed that MUFAs are mainly transported through the TG content of these lipoproteins, while PUFAs are mainly transported through the phospholipids or cholesterol ester content of lipoproteins [66]. We identified several associations for CpG methylation in *SCD, FADS1/2, SLC7A11, TXNIP,* and *PHGDH* with PUFAs. However, in line with previous studies, those in the *FADS* region appear to have a complex (epi)genetic architecture [37, 67–70]. The only CpG site showing associations with PUFAs, and additionally with the respective gene transcript, metabolic measure ratios, and disease endpoints was cg16246545 in *PHGDH*. Since we found no associations between methylation at cg16246545 and lipoproteins, but we did see that methylation at this site was associated with omega-6 PUFAs such as linoleic acid (LA), PUFAs and DNA methylation might have an interrelation that does not involve lipoproteins. Omega-6 PUFAs intake has been associated with changes to DNA methylation and metabolic alterations [71]. Additionally, the inhibition of *PHGDH* induces changes in DNA methylation and broad changes in metabolism such as alterations in nucleotide metabolism [72, 73]. Higher LA consumption might thus be related to methylation of cg16246545. As we also found an association of this site with gene expression, this may be a pathway through which LA consumption leads to adverse metabolic outcomes such as obesity. Previously, we demonstrated an association between *PHGDH* transcription and a CpG site only 50 bp downstream from cg16246545 [25, 74]. We hypothesize that not only a single CpG site, but rather a bigger genetic region which is overlapping active histone marks, contributes to the functional relevance of cg16246545. Although we do not confirm negative associations of methylation at cg16246545 with serum total TG levels, we found novel associations with omega-6 FAs, fatty acids that are involved in regulation of TG levels [18].

The major strength of this work is the detailed information presented by the serum metabolic measures relating to the sizes, lipid compositions, and lipid concentrations of the lipoprotein subclasses. All CpG sites found to be associated with metabolic measures have additionally been associated with many more cardiometabolic conditions such as liver enzymes and hepatic steatosis [75] among others, thus extending the relevance of our findings. The generalizability of our results to a broad range of populations seems plausible, as our study uses population-based cohorts with different ethnic backgrounds [76]. We have confidence in our results owing to the overall large sample size of our study, the high percentage of replicated associations in the largest replication cohort (LOLIPOP), and the consistent directions of effect and Pearson correlations with the discovery cohort coefficients across all replication cohorts. However, NFBC1966 and YFS replication cohorts showed smaller effect sizes than those found in the discovery cohort, perhaps due to the younger age and healthier status of NFBC1966 and YFS participants. Additionally, cross-sectional studies do not readily provide information on causation in the context of DNA methylation, although recent studies imply an effect of lipid levels on DNA methylation rather than vice versa [24], and DNA methylation has often been considered a biomarker rather than a predictor [20, 24, 25, 29, 31, 32]. Mendelian randomization studies involving meta-analyses of studies of larger sample sizes than investigated here are needed to unravel the causal structures of the associations presented in this work [30]. Further in vitro and/or in vivo studies could also clarify causes and functional consequences of lipid-related DNA methylation alterations. Another limitation is the fact that DNA methylation was analyzed in DNA extracted from whole blood, a mixture of different cell types, while the investigated metabolic measures largely originate from metabolic processes in the liver, muscle, and adipose tissue. Nevertheless, blood represents an easy-to-obtain human tissue that can be used for predictive, prognostic, and intervention biomarkers, and so its detailed investigation is certainly warranted.

Our findings could potentially be used as part of a multifaceted approach that incorporates genetic data, epigenetic data, and genetic–epigenetic interactions for complex disease prediction [77], hence offering future researchers a building block for developing biomarkers for dyslipidemia and other cardiometabolic diseases.

## Conclusion

In summary, serum metabolic measures were found to be associated with the methylation levels of interrelating genes involved in lipid metabolism and cardiometabolic disturbances. We observed that DNA methylation is linked to the sizes, lipid compositions, and lipid concentrations of apolipoprotein B-containing lipoprotein and HDL subclasses. No evidence of a link between DNA methylation and PUFAs involving lipoproteins was obtained. Our results provide in-depth insights into previous metabolic trait-DNA methylation associations based on total concentrations of serum lipids and indicate a complex regulation of the human metabolism possibly closely interrelated with epigenetic processes. We demonstrate the power of detailed metabolic measure profiling in large population-based cohorts to improve the molecular understanding of dyslipidemia and related disease mechanisms. Further studies are needed to clarify underlying functional mechanisms and identify pharmaceutical interventions for cardiometabolic disturbances.

## Methods

### Study design

The aim of this study was to identify the association of DNA methylation and a set of 226 mostly lipid-related NMR-measured serum metabolic measures. The design of the study comprised discovery and replication stages with subsequent follow-up analyses of the CpG sites associated with metabolic measures (Fig. 3). The discovery stage consisted of an EWAS of metabolic measures from the KORA cohort with subsequent validation of robust associations in the LOLIPOP, NFBC1966, and YFS replication cohorts. In the follow-up studies we assessed potential genetic confounding of the obtained associations, whether the associations varied between the sexes, and whether the CpG sites were associated with metabolic measure ratios, gene expression, and disease endpoints. Follow-up studies were performed in the KORA cohort using only those CpG sites that showed replicated associations with metabolic measures.

### Discovery cohort

The Cooperative Health Research in the Region of Augsburg (KORA) study is a series of independent population-based epidemiological surveys and follow-up studies of participants living in the region of Augsburg, Southern Germany. The studies have been conducted according to the principles expressed in the Declaration of Helsinki. The KORA F4 study, a seven-year follow-up study of the KORA S4 survey (examined 1999–2001), was conducted between 2006 and 2008. The standardized examinations applied in the survey have been described in detail elsewhere [78]. A total of 3080 subjects with ages ranging from 32 to 81 years participated in the examination. Anthropometric and serum measures were measured concurrently. Aliquots of whole blood were stored at − 80 °C for extraction of genomic DNA. In a random subgroup of 1802 KORA F4 subjects DNA methylation patterns were analyzed. Of the 1790 subjects who also had serum metabolic measures, 36 had detection rates of less than 95% over all measures and were eliminated from further analysis. Eight individuals were further eliminated due to non-fasting status at the time of blood sampling and 84 due to lack of valid methylation data, leaving a final sample size of 1662 subjects. Clinical phenotypes were defined as follows: type 2 diabetes (T2D), self-report, or intake of glucose-lowering medication, excluding metformin; hypertension, ≥ 140/90 mmHg, or intake of anti-hypertensive medication; obesity, BMI ≥ 30; previous myocardial infarction (MI), self-report.

### Replication cohorts

The London Life Sciences Prospective Population Study (LOLIPOP) is a prospective cohort study of ~ 28000 Indian Asian and European men and women, recruited from the lists of 58 General Practitioners in West London, UK, between 2003 and 2008. In 4060 samples of Indian-Asian subjects, anthropometric and serum measures were measured concurrently, and aliquots of whole blood were stored at − 80 °C for extraction of genomic DNA. DNA methylation was quantified in a subset of 2805 participants.

The Northern Finland Birth Cohort 1966 (NFBC1966) is a prospective population-based birth cohort, in the two northernmost provinces of Finland (*N* = 12055) with children whose expected date of birth was in the year 1966. In 1997–1998, a postal questionnaire on health, social status, and lifestyle was sent to the living cohort members, and those living in the original target area or in the capital area were invited for a clinical examination, including blood sample collection. Aliquots of whole blood were stored at − 80 °C for later extraction of genomic DNA. DNA methylation patterns were analyzed for 807 subjects randomly selected.

The Cardiovascular Risk in Young Finns Study (YFS) is an ongoing multicentre Finnish longitudinal population study sample on the evolution of cardiovascular risk factors from childhood to adulthood. The study began in 1980, when 3596 participants between the ages of 3 and 18 were randomly selected from the national population registers. Anthropometric and serum measures were measured concurrently, and aliquots of whole blood were stored at − 80 °C for extraction of genomic DNA. In a subsample of 184 individuals randomly assigned from a follow-up in 2011, DNA methylation patterns were determined.

### DNA methylation quantification

DNA methylation was quantified in bisulfite-converted genomic DNA from whole blood samples of all participants in both the discovery (*N* = 1662) and replication cohorts (*N* = 3752), using the Infinium HumanMethylation450 BeadChip (450 K BeadChip) (Illumina Inc, San Diego, CA, USA) in the discovery and replication cohorts. Further details on processing of the methylation data can be found in the supplemental methods (Additional file 4: Supplemental Results and Methods).

### Serum metabolomics

Participants of all cohorts were in a state of fasting when blood samples were collected. Metabolite detection and quantification were performed on a high-throughput nuclear magnetic resonance (NMR) spectroscopy-based platform (Nightingale Ltd, Helsinki, Finland) [79, 80]. A total of 228 serum metabolic measures were assessed, and after data quality control 226 remained: 147 directly measured, mostly given in concentration units, and 79 derived ratios, mostly given in percentage, such as the ratios of specific types of lipids to total lipids in lipoprotein subclasses. The metabolic measures included six VLDL-, one IDL-, three LDL-, and four HDL-lipoprotein size-subclasses. Each lipoprotein size-subclass was measured for concentration and composition of phospholipids, total and free cholesterol, cholesterol esters, triglycerides, and total lipids. Additionally, two apolipoproteins, eight fatty acids, eight glycerides and phospholipids, nine cholesterols, nine amino acids, one inflammatory marker, and ten small molecules involved in glycolysis, citric acid cycle, or urea cycle were measured. Further details on sample preparation and the metabolic measure data can be found in the supplemental methods (Additional file 4: Supplemental Results and Methods).

### Statistical analysis

#### Epigenome-wide association studies: discovery and meta-analysis

The discovery stage in the KORA F4 (*N* = 1662) cohort was made up of 226 epigenome-wide association studies, one per investigated metabolic measure passing quality control (Additional file 1: Table S1). Specifically, for each metabolic measure, 468151 linear regression models were examined, one per CpG site. Each model used the natural logarithm of the metabolic measure as the dependent variable and technically adjusted beta values (i.e., proportion of methylation at the given CpG site) and covariates (detailed in Additional file 4: Supplemental Results and Methods) as explanatory variables. The covariates used in the linear models as potential confounders were age, sex, body mass index (kg/m^2^), c-reactive protein (mg/l), HbA1c (%), smoking status (current smoker, ex-smoker, or never smoker), alcohol consumption (g/day), lipid-lowering drug use (yes/no), presence of hypertension (yes/no), history of self-reported myocardial infarction (yes/no), level of physical activity (high/low), total white blood cell count (/nl), and proportions of white blood cell types as estimated using the Houseman method [81]. Statistical significance was determined using a Bonferroni-corrected threshold (*p* < 0.05/(468151 × 226) ≈ 4.7e−10). Following the discovery, we ran sensitivity analyses to examine the model assumptions and the robustness of the results (Additional file 4: Supplemental Results and Methods). We then tested for replication of those significant CpG-metabolic measure pairs showing robustness using a meta-analysis of the results of the three participating replication studies (LOLIPOP, *N* = 2805; NFBC1966, *N* = 771; YFS, *N* = 176; statistical significance *p* < 0.05/274 ≈ 1.8e−4).

#### Genetic effects analysis

Multi-omics analyses were performed in the discovery cohort for associated CpG sites. Conditional analyses were performed to investigate whether genetic variation (single nucleotide polymorphisms, SNPs) within 1 Mb of the CpG sites could drive the relationships between the metabolic measures and methylation. For each CpG-metabolic measure pair, associated SNPs within 1 Mb of the CpG site were added singly to the models to determine the effect of the SNP on the association. Full details are given in the supplemental methods (Additional file 4: Supplemental Results and Methods).

#### Gene expression analysis

To study the interplay between the identified CpG sites and gene expression we examined associations with gene expression probes lying within 1 Mb of the significant CpG sites and extended these investigations to tissues beyond whole blood using data extracted from the ArrayExpress database [82] for both subcutaneous fat (TwinsUK study, ArrayExpress references E-TABM-1140, and E-MTAB-1866 [83, 84]) and liver (Karolinska Liver Bank cohort ArrayExpress reference E-GEOD-61279 [85]). Statistical significance was determined in the discovery cohort as *p* < 0.05/480 ≈ 1.0e−4; *p* < 0.05/521 ≈ 9.6e−5 for subcutaneous fat; and *p* < 0.05/271 ≈ 1.8e−4 for liver; based on the total number of CpG-expression probe pairs examined per tissue. Results from the BIOS QTL browser (FDR < 0.05), a database presenting whole blood expression-methylation associations [41, 86], were integrated into significant CpG-transcript associations. Additional details on the gene expression analysis can be found in the supplemental methods (Additional file 4: Supplemental Results and Methods).

#### Sex interaction analysis

Sex interaction analysis was performed in the discovery cohort for each replicated CpG site-metabolic measure association, excluding those involving CpG sites from the *FADS* region. The models were identical to the discovery models, but with a “sex × methylation” interaction term (males as reference sex). Statistical significance for the interaction coefficient was judged at a Bonferroni-corrected threshold of *p* < 0.05/148 ≈ 3.4e−4.

#### Associations with serum metabolic measures ratios implicated in different pathways

In an attempt to identify specific steps of metabolic pathways that might be linked to DNA methylation in the discovery cohort KORA F4, we assessed association of CpG sites with additional ratios beyond those provided by the platform, using linear regression and adjusting for the same covariates used in the discovery EWAS. We calculated additional ratios related to the lipolysis, proteolysis, glycolysis, and ketogenesis pathways. Only associated metabolic measures and CpG sites from the replicated results were included. Statistical significance was determined as *p* < 0.05/(60 * 12) ≈ 6.9e−5. The tests for the associations of the 12 CpG sites with 30 transcripts of proteins directly involved in lipoprotein metabolism such as enzymes, transfer proteins, and lipid transporters was performed as for the gene expression analysis described for KORA F4 above and in the supplemental methods, but only looking additionally at probes within 500 bp, rather than 1 Mb. Statistical significance was determined as p < 0.05/(62 * 12) ≈ 6.7e−5. Further details on the selection criteria of ratios and transcripts can be found in the supplemental methods (Additional file 4: Supplemental Results and Methods).

#### Associations with clinical phenotypes

We next determined whether replicated CpG sites were associated with prevalent type 2 diabetes (T2D, self-report, or intake of glucose-lowering medication, excluding metformin; *N* = 148 cases, 1516 controls), hypertension (≥ 140/90 mmHg, or intake of anti-hypertensive medication; *N* = 757 cases, 901 controls), obesity (BMI ≥ 30; *N* = 497 cases, 1159 controls), or previous myocardial infarction (MI, self-report; *N* = 59 cases, 1602 controls) in the discovery cohort. For each CpG site and each outcome, we performed logistic regression adjusted for all covariates as in the discovery analysis (excluding HbA1c for diabetes and BMI for obesity) (model 1). To explore the effects of lipid-lowering drugs on the relationships, we ran two models: one without this covariate (model 1) and one with (model 2). Statistical significance was determined at a Bonferroni-corrected threshold of *p* < 0.05/(4 * 12) ≈ 1.0e−3.

Additional details on the data preparation, statistical analysis of the discovery and meta-analysis, multi-omics analyses, and associations with ratios and clinical phenotypes can be found in the supplemental methods (Additional file 4: Supplemental Results and Methods). All statistical significance was determined using Bonferroni-corrected thresholds based on family-wise type I error rates of 0.05 and the number of relevant tests, except where noted.

## Supplementary information

**Additional file 1: Table S1.** List of the metabolic measures (*N* = 228) assessed in all cohorts.

**Additional file 2: Table S2.** Epigenome-wide association study (EWAS) of metabolic measures. Presented are CpG site-metabolic measurement pairs statistically significantly associated in the KORA F4 discovery study. Results for the discovery analysis in KORA (“KORA”), the sensitivity analysis in KORA (“_KORA_Sens”), LOLIPOP (“_LOLIPOP”), NFBC1966 (“_NFBC”), and Young Finns (“_YFS”) studies, as well as the results for the meta-analysis (“_MA”) of these three replication studies, are presented. The scaled coefficients of the sensitivity analysis in KORA (“Coef_KORA_scaled”, i.e., both the log-transformed metabolite measure and methylation beta value were z-transformed prior to analysis) are also presented. The coefficients and *p* values for the discovery KORA analysis were calculated based on the results of 10 MICE imputed datasets and combined using the commands pool.scalar and micombine.chisquare, from the R packages mice and micetools, respectively. Discovery (KORA F4) significance based on *p* value < 4.73e−10; meta-analysis significance based on *p* value < 1.80e−4 (based on 274 pairs tested for replication). Gene, CHR, and Pos: gene, chromosome, and position annotation for the CpG site taken from the Illumina 450 K annotation file; Coef: coefficient of the CpG site from the regression analysis; SE: standard error of the coefficient; P: *p* value for the regression coefficient; *N*: number of observations; P_Bonf: Bonferroni-corrected *p* value for the given analysis; Explained_variance_KORA: percentage of explained variance of the log-transformed metabolic measure by the CpG; Stat_Sig_MA: statistically significant in the meta-analysis of the three replication studies. Unless otherwise specified, all coefficients are change in natural log-transformed metabolite measurement unit (as given in Additional file 1: Table S1) per unit increase in methylation (beta value on 0–1 scale).

**Additional file 3: Table S3.** Summary statistics comparing the 274 KORA-significant associations across the replication cohorts. Results are given as total number (proportion) or as *p* values calculated using the binomial distribution (*n *= number of valid models in the study), where the probability for a single Bernoulli trial is given as *p = 0.5, **0.05, ***0.05/274. The number of valid models is the number of pairs for which results were available.

**Additional file 4: Supplemental Results and Methods.** In the Supplemental Results section a detailed comparison of the results obtained across cohorts can be found. In the Supplemental Methods section a detailed description of data processing for each of the population based cohorts can be found, as well as detailed specification on statistical and multi-omics analyses.

**Additional file 5: Table S4.** Replication tables. Replicated associations are displayed by CpG site, CpG gene location, metabolite (metabolic measure), and metabolite type (metabolic measure type). A total of 274 significant, robust associations in the discovery cohort (KORA F4) were found.

**Additional file 6: Figure S1.** Pearson correlations between metabolic measures associated with DNA methylation, KORA F4 data. Correlations among DNA methylation-associated metabolic measures are shown. All metabolic measures found associated with methylation in the discovery cohort are included.

**Additional file 7: Figure S2**. Explained variance of the first 30 principal components of the metabolite measure principal component analysis, performed in the discovery cohort KORA F4.

**Additional file 8: Figure S3.** Presented are the first 8 principal components (PCs) of the metabolite data in KORA F4 (after scaling of the individual metabolites) coloured according to sex, intake of lipid-lowering drugs, and smoking habits. “Expl. Var” is the variance explained by the given PC. Some clustering is observed for sex within the first 2 PCs, but no other obvious clusters emerge for any of the other phenotypes or PCs.

**Additional file 9: Table S5.** Results of the EWAS of metabolite measure principal components. Presented are the statistically significant (Bonferroni-adjusted *P* < 1.34e−8) results of the EWAS of the first 8 principal components of the metabolite measures data in the discovery cohort KORA F4. CHR: annotated chromosome of the CpG site; Pos: annotated chromosomal position of the CpG site; Coef: coefficient of the CpG site in the regression model; SE: standard error of the coefficient; P: *p* value; PC_explained_var: Explained variance of the metabolite principal component.

**Additional file 10: Table S6.** Results of look-ups in two epigenome-wide association study (EWAS) catalogues, the EWAS Atlas [39] (Source: “EWAS_Atlas”) and the EWAS Catalog [40] (Source: “EWAS_Cat”). Shown are the catalogue results (significantly associated CpGs and traits) for all CpGs from our replicated metabolic measure-CpG associations (16 CpG sites total). Chromosome (CHR) and chromosomal position (Pos) taken from the Illumina HumanMethylation450 v1.2 Manifest File, available from the Illumina website.

**Additional file 11: Table S7.** Genetically influenced CpG site-metabolic measure associations. Results for the *cis*-SNP analysis are presented. “Coef_discovery” and “P_discovery”: coefficient and *p* value for the discovery analysis for the given CpG site-trait pair. “Count_conf_SNPs”: number of SNPs which, when added singly to the metabolic measure-CpG site regression model, cause the pair to lose its statistical significance, as defined by the discovery threshold (*p* = 4.73e−10). “Coef_adj” and “p_adj”: coefficient and *p* value of the CpG in the model with the addition of the SNP causing the greatest effect (largest change in *p* value). “top_SNP”: name of the SNP causing the largest effect. “Loses_significance” indicates whether the addition of any SNP to the CpG-metabolic measure regression model causes the association to lose significance (i.e., “Count_conf_SNPs” > 0). “Probable_SNP_confounding” indicates the addition of at least one single SNP to the model renders the association insignificant and drastically alters the results, indicated likely SNP confounding by 1 or more SNPs. NAs within the table indicate the absence of a SNP fulfilling the requirements of our conditional analyses for the CpG-metabolic measure pair (i.e., the absence of a SNP being associated with both the CpG site and the metabolic measure). CHR: chromosome, pos: position, UCSC_RefGene_Name_CpG: annotated gene name of the CpG site, all as given by the Illumina manifest file. N: number of observations in the model incorporating the SNP. All coefficients are change in natural-log transformed metabolite measurement unit (as given in Additional file 1: Table S1) per unit increase in methylation (beta value on 0–1 scale). *Drastic increase (factor > 100) of *p* value after addition of the SNP to the regression model.

**Additional file 12: Table S8.** Results of gene expression analysis of CpG sites associated with metabolic measures. Displayed are the FDR (Benjamini-Hochberg, < 0.05) statistically significant associations between metabolic measure-associated CpG sites and expression transcripts in *cis* in whole blood (KORA F4, 480 CpG-transcript pairs examined), subcutaneous fat (TwinsUK study, 521 pairs examined) and liver (Karolinska Liver Bank cohort and the Dutch tissue cohort MORE/BBMRI obesity cohort, 271 pairs examined). FDR-adjusted *p* values were calculated for each tissue separately. “Probe ID” and “Transcript_annotated_gene”: transcript ID and annotated gene from the Illumina annotation files. “Distance”: distance between the CpG and the transcript according to the annotation files. CHR: chromosome; Distance: distance between the CpG and the transcript based on positions given in the annotation files; Coef: beta-coefficient; P: *p* value; FDR: FDR-corrected *p* value; Bonf_P: Bonferroni-corrected *p* value; N: number of observations in the model; “replicated_BIOS”: whether the association (where the CpG is matched directly, but the transcript is matched by annotated gene only), with consistent direction of effect, is found in the FDR < 0.05 significant BIOS QTL database. An “-” indicates the CpG-gene expression pair is not found in the FDR < 0.05 significant results in BIOS. All coefficients are change in log2- transformed expression intensity per unit increase in methylation (beta value on 0–1 scale), except for subcutaneous fat, which is correlation assessed using the R-package rmcorr.

**Additional file 13: Table S9.** Results of the sex interaction analysis. Results for all 148 replication CpG site-metabolic measure associations are shown for models identical to the discovery, but with additional “sex × methylation” interaction term. Presented are the obtained coefficient (interaction_coef) for the term of the interaction between methylation and sex (0: male, 1: female), standard error of the coefficient (interaction_SE), *p* value of the coefficient (interaction_P), the Bonferroni-corrected *p* value of the coefficient (interaction_bonf_p), the methylation-metabolite measure coefficient for males (coef_CpG_Met_male), with the calculated females coefficient (coef_CpG_Met_female, i.e., coef_CpG_Met_male + interaction_coef), and number of observations for the model (N). No interaction coefficient results pass a Bonferroni-corrected *p* value threshold of 0.05/148 ~ 3.4e−4 for statistical significance. All coefficients are change in natural-log transformed metabolite measurement unit (as given in Additional file 1: Table S1) per unit increase in methylation (beta value on 0–1 scale).

**Additional file 14: Table S10.** Results of the associations with additional metabolic measure ratios as proxies of enzymatic activity or linked to metabolic disease. Results for all 12 replicated metabolic measure-associated CpG sites associations are shown. Presented are the coefficient (Coef) of the CpG site from the regression analysis, standard error of the coefficient (SE), *p* value of the coefficient (P), Bonferroni-corrected *p* value (Bonf_P) and number of observations for the model (N). A Bonferroni-corrected *p* value threshold of p < 0.05/(60 * 12) ≈ 6.9e−5 is used for statistical significance. All coefficients are change in difference of natural-log transformed metabolite measurement units (as given in Additional file 1: Table S1, i.e., log(metabolite 1) − log(metabolite 2)) per unit increase in methylation (beta value on 0–1 scale).

**Additional file 15: Table S11.** Results of the associations between CpG sites and expression of transcripts of genes codifying for enzymes or proteins directly involved with lipoprotein metabolism. Results are shown for those pairs passing a false discovery rate (Benjamini-Hochberg) threshold of FDR < 0.05. Presented are the CpG sites and expression probe IDs; the annotated genes of the CpG site and expression probe; the chromosomes of the CpG site and expression probe; the chromosomal location of the CpG site; the distance between the CpG site and expression probe (where Inf indicates they are on different chromosomes); the coefficient (Coef) of the CpG site from the regression analysis; standard error of the coefficient (SE); *p* value of the coefficient (P); Bonferroni-corrected *p* value (Bonf_P); FDR-corrected *p* value (FDR); number of observations for the model (N); and whether the association is significant at Bonferroni-corrected threshold (based on 12 CpGs × 62 expression probes: *p *< 0.05/(12 * 62) =6.7e−5 ). All coefficients are change in log2- transformed expression intensity per unit increase in methylation (beta value on 0–1 scale).

**Additional file 16: Table S12.** Results of the investigation into associations between metabolic measures-related CpG sites and clinical outcomes. Listed are the CpG site-clinical outcome (previous myocardial infarction, prevalent type 2 diabetes, obesity and prevalent hypertension) results for both model 1: logistic regression model with clinical outcome as dependent variable and technically adjusted methylation value as independent variable, adjusted for the following covariates: age, sex, BMI, C-reactive protein levels, hemoglobin A1c levels (except for diabetes model), history of myocardial infarction (except for the myocardial infarction model), smoking status, current hypertension (except for the hypertension model), physical activity, white blood cell count and estimated proportions of white blood cell type; or model 2, which is additionally adjusted for intake of lipid-lowering drugs. P: *p* value of the association; OR: odds ratio for a 1 standard deviation increase in methylation; CI: confidence interval; FDR: false discovery rate *p* value based on the Benjamini–Hochberg method applied to each outcome and model separately; M1: model 1; M2: model 2. *Association investigated using the KORA F4 data in [15]; **Association investigated using the KORA F4 data in [25].

**Additional file 17: Table S13.** ROC curve analysis for significant CpG-outcome associations: Presented are the areas under the curve (AUC) for the receiver operating characteristic (ROC) curve analysis for each outcome-CpG pair for which there exists a statistically significant association for either M1 or M2 (Table 3). Presented are the results for the intercept (mean) model and the model with the CpG sites and no other covariates; for M1 without and with the CpG site; and for M2 without and with the CpG site. Presented are the AUCs for the respective ROCs, and a *p* value for the null hypothesis that the addition of the CpG site to the model has no effect on the predictive performance of the model. The *p* value was determined using the R package pROC [87], command roc.test, method “bootstrap”. The analysis was run in the KORA F4 dataset, and, to ensure comparability of the results, the ROC curves were generated using individuals with no missing values in any of the outcomes or covariates, and the methylation data were mean imputed.

**Additional file 18: Figure S4.** ROC curves for significant CpG-outcome associations: Presented are the receiver operating characteristic (ROC) curves for each outcome-CpG pair for which there exists a statistically significant association for either M1 or M2 (Table 3). The red line is the ROC curve for the model without the CpG, and the green line is the model with the CpG. Presented are also the areas under the curve (AUC) for the respective ROCs, and a *p* value for the null hypothesis that the addition of the CpG to the model has no effect on the predictive performance of the model. The *p* value was determined using the R package pROC [87], command roc.test, method “bootstrap”. The analysis was run in the KORA F4 dataset, and, to ensure comparability of the results, the ROC curves were generated using individuals with no missing values in any of the outcomes or covariates, and the methylation data were mean imputed.

**Additional file 19: Table S14.** Inflation factors for each epigenome-wide association analysis. Presented are the genomic inflation factors (lambda) for all 226 EWAS run in the discovery analysis in KORA F4. All inflation factors were calculated using complete case analysis.

## Data Availability

The publicly available datasets analyzed during the current study included subcutaneous fat data (TwinsUK study) and liver data (Karolinska Liver Bank cohort) which are available in the following repositories: ArrayExpress reference E-TABM-1140 https://www.ebi.ac.uk/arrayexpress/experiments/E-TABM-1140/. ArrayExpress reference E-MTAB-1866 https://www.ebi.ac.uk/arrayexpress/experiments/E-MTAB-1866/. ArrayExpress reference E-GEOD-61279 https://www.ebi.ac.uk/arrayexpress/experiments/E-GEOD-61279/.

## Abbreviations

DHA: Docosahexaenoic acid
DNA: Deoxyribonucleic acid
EWAS: Epigenome-wide association study
FA: Fatty acids
HbA1c: Hemoglobin A1c
HDL: High-density lipoproteins
IDL: Intermediate-density lipoproteins
KORA: Cooperative Health Research in the Region of Augsburg
LA: Linoleic acid
LDL: Low-density lipoproteins
lncRNA: Long non-coding RNA
LOLIPOP: London Life Sciences Prospective Population Study
Mb: Mega-base pairs
MI: Myocardial infarction
miRNA: Micro-ribonucleic acid
mRNA: Messenger ribonucleic acid
MUFA: Monounsaturated fatty acids
NFBC1966: Northern Finland Birth Cohort 1966
NMR: Nuclear magnetic resonance
PUFA: Polyunsaturated fatty acids
RCT: Reverse cholesterol transport
SNP: Single nucleotide polymorphisms
T2D: Type 2 diabetes
TG: Triglycerides
VLDL: Very low-density lipoproteins
YFS: Cardiovascular Risk in Young Finns
