# Supplementary material for: DNA methylation and lipid metabolism: an EWAS of 226 metabolic measures
Source: Clin Epigenetics. 2021 Jan 7;13:7. doi: 10.1186/s13148-020-00957-8 (PMC7789600; doi:10.1186/s13148-020-00957-8)
Supplement: Supplementary file 4 — Additional file 4: Supplemental Results and Methods. In the Supplemental Results section a detailed comparison of the results obtained across cohorts can be found. In the Supplemental Methods section a detailed description of data processing for each of the population based cohorts can be found, as well as detailed specification on statistical and multi-omics analyses. [file 13148_2020_957_MOESM4_ESM.docx]

**DNA methylation and lipid metabolism: an EWAS of 226 metabolic measures**

**Supplemental Results and Methods**

**Supplemental results**

*Comparison across cohorts*

Supplemental table 3 (Additional File 3: Supplemental Table 3) presents the cross-study comparison of the results of the 274 significant KORA associations. At a Bonferroni-corrected p-value threshold (p < 0.05 / 274 = 1.8e-4, family-wise error rate of 0.05) and consistent direction of effect (same effect sign as KORA discovery), LOLIPOP replicated 95% of the associations, NFBC 1966 7% and Young Finns 7%. At a nominal level (p < 0.05) and consistent direction of effect, LOLIPOP replicated 99.6% of the associations, NFBC 1966 54% and Young Finns 30%. The Pearson correlations with the KORA coefficients were 1.0, 0.9 and 0.8 for LOLIPOP, NFBC 1966 and Young Finns, respectively (p < 2.2e-16 for all). Across all 274 associations, LOLIPOP had the same direction of effect as KORA for 99.6% of the pairs, NFBC 1966 for 97%, and Young Finns for 88%. To see that the results for NFBC 1966 and Young Finns were not driven by the nominally significant results, we note that for those pairs with p > 0.05 in the respective study, the NFBC 1966 still had consistent direction of effect with KORA for 94% of the pairs, and Young Finns with 83%. This indicates that in general, even if a pair does not reach nominal significance, it is still highly likely to have the same direction of effect as KORA.

It is noted that in terms of magnitude of effect, LOLIPOP has 54% of the effect sizes greater in magnitude than KORA (p-value = 0.25 for a null hypothesis of equal probability of larger or smaller effect size based on binomial distribution (n=274, p = 0.5)), indicating roughly equal distributions of effect sizes for the two studies. For NFBC 1966 (12%, p = 3.3e-40) and Young Finns (37%, p = 1.4e-5), however, there was a statistically significant trend for smaller effect sizes compared to KORA, particularly for NFBC 1966.

**Supplemental methods**

All statistical analyses were performed using R (v3.0.2 or later), except where noted.

*Population-based cohort studies*

*KORA F4 Study - Discovery cohort*

*DNA methylation data preprocessing*

Genome-wide DNA methylation patterns of whole blood were analyzed using the Infinium HumanMethylation450 BeadChip Array (Illumina) as described earlier (1). Raw DNA methylation data were extracted with Illumina Genome Studio (version 2011.1), methylation module (v1.9.0), and processed using R (v3.0.1) following the CPACOR pipeline of Lehne et al. (2) including exclusion of 65 SNP probes and background correction using *minfi* (3). Probes were set to missing if the detection p-value≥0.01 or the number of beads < 3. Samples were excluded if the detection rate was ≤ 0.95. Quantile normalization was performed on intensity values separated by colour channel, probe type and M/U subtypes. The resulting methylated and unmethylated signals were used to calculate β-values, a measure of percent methylation between 0 and 1.

To further eliminate technical effects, the principal components (PCs) of the intensities of the (non-negative, autosomal) control probes were calculated. For each CpG site, the residuals of a linear regression model with β-value as outcome and the first 20 control probe PCs as covariates were used as the “technically adjusted β-values” in all analysis. Methylation outliers, defined as those technically adjusted β-values more than 5 standard deviations from the mean for each CpG site, were set to missing. CpG sites annotated to the X or Y chromosomes or with greater than 20% missing values were eliminated, leaving 468151 CpG sites, with 0.7% missing values total.

Examination of the top principal components of the methylation data revealed no obvious outlying individuals to be excluded.

*Metabolomics data preprocessing*

Metabolic measures were performed on a nuclear magnetic resonance (NMR) spectroscopy-based platform (total number of metabolic measures = 228) described elsewhere (4, 5). Lipoprotein measured size-subclasses were classified as follows: chylomicrons and very large VLDL (average particle diameter > 75 nm), very large VLDL (average particle diameter of 64.0 nm), large VLDL (53.6 nm), medium VLDL (44.5 nm), small VLDL (36.8 nm) and very small VLDL (31.3nm); IDL (28.6 nm); large LDL (25.5 nm), medium LDL (23.0 nm) and small LDL (18.7 nm); very large HDL (14.3 nm), large HDL (12.1 nm), medium HDL (10.9 nm) and small HDL (8.7 nm).

Computational strategies of metabolite identification and quantification from the NMR spectra are described by Inouye et al (4). Of the 228 metabolic measures, two measures (glycine and glycerol) had missing values or zero values in greater than 10% of the samples and were removed.

The natural logarithm was taken of all the metabolic measures to achieve normal distributions, firstly setting the zero values to half the lowest non-zero value seen for that metabolic measure. Outliers, defined as those concentrations further than 5 standard deviations from the mean for that metabolic measure, were set to missing. The total number of missing values was then 3559 (0.9%) over all 226 metabolic measures, with a median of three missing values per metabolic measure and a maximum of 6% missingness for “cholesterol esters to total lipids ratio in chylomicrons and extremely large VLDL”.

*Covariate data preprocessing*

The covariates used in the linear models as potential confounders were age, sex, body mass index (kg/m2), c-reactive protein (mg/l), HbA1c (%), smoking status (current smoker, ex-smoker or never smoker), alcohol consumption (g/day), lipid-lowering drug use (yes/no), presence of hypertension (yes/no), history of self-reported myocardial infarction (yes/no), level of physical activity (high/low), total white blood cell count (/nl), and proportions of white blood cell types as estimated using the Houseman method (6). Over all 1662 individuals there were a total of 20 missing values within the covariates.

*Imputation of missing data for EWAS discovery*

For the epigenome-wide association discovery, imputation was performed for all missing values for the methylation, metabolic measure and covariate data. For the methylation data mean imputation was performed using the omicABEL software (7). Multiple imputation by chained equations (8) was performed for the metabolic measures and covariate data, to create 10 complete covariate and metabolic measure datasets. For the analysis following the EWAS discovery, complete cases were used in a single, non-imputed analysis, except where indicated.

*LOLIPOP study - Replication cohort*

*Methylation data preprocessing*

DNA methylation was quantified in bisulfite converted genomic DNA from whole blood, using the Illumina Infinium HumanMethylation450 array in 4,060 samples. DNA methylation was quantified on a scale of 0-1, where 1 represents 100% methylation.

Preprocessing and quality control criteria have been described previously (2). Briefly, raw signal intensities were retrieved using the function readIDAT of the R package minfi, version 1.6.034, from the Bioconductor open source software (9), followed by background correction with the function bgcorrect.illumina from the same R package. Detection P values were derived using the function detectionP as the probability of the total signal (methylation + unmethylated) being detected above the background signal level, as estimated from negative control probes. Signals with detection P values ≥ 10e-16 were removed. Samples with less than 95% CpG sites providing a signal were subsequently excluded from the data set. To reduce non-biological variability between observations, data were quantile normalized with the function normalizeQuantiles of the R package limma, version 2.12.035, from Bioconductor, separately in six probe categories based on probe type and colour channel.

In order to account for technical and biological confounders we performed Control Probe Adjustment (2) and estimated proportions of white blood cell types as described by Houseman et al. (6). These proportions were subsequently used as covariates in the model to avoid cell type confounding.

*NFBC study* *- Replication cohort*

DNA was extracted and DNA methylation patterns were analyzed for 807 subjects using the Infinium HumanMethylation450 BeadChip (Illumina). Methylation data was preprocessed according to the CPACOR EWAS analysis pipeline phases 1-4 (2). For DNA methylation marker calling we used a detection P value threshold of <10e-16. A call rate filter of 95% was applied to the all autosomal Illumina probes yielding 459,378 probes for association testing. 67 samples were excluded due to low marker call rate (<95%). 7 samples were excluded for gender inconsistency; one sample for globally outlying DNA methylation values (1st PC score of the DNA methylation values outside mean +/- 4SD).

*Young Finns Study - Replication cohort*

*Methylation data preprocessing*

In brief, methylation signal data was preprocessed as a methylumiset object using the R software (R >= 2.15.3) with array-specific algorithms implemented in the R packages wateRmelon and BMIQ as previously described (10). The resulting β values ranged linearly from 0 (non-methylated, 0%) to 1 (completely methylated, 100%). The quality of DNA samples and methylation data was carefully ensured by standard examinations with principal component analysis (PCA) and visualizations with density plots, boxplots, and dotplots. Three of the YFS samples were excluded due to atypically low probe intensities compared with control probe intensities. The first 20 control probe principal components were calculated as per KORA and the "technically adjusted β-values" were used as the beta values in all models. Various measures of age acceleration, estimates of blood cell counts and predicted gender (based on the DNAm levels of X chromosomal markers) were obtained using the online DNA Methylation Age Calculator (<https://dnamage.genetics.ucla.edu/>) (11) with non-normalized raw β values as an input and with the "Normalize Data" and "Advanced Analysis for Blood Data" options. Based on the recommendations in the online tutorial, the following cell counts based on the Houseman and Horvath methods were used as covariates in the present analyses: CD8.naive, CD8pCD28nCD45RAn, PlasmaBlast, CD4T, NK, Mono, Gran. Two samples were excluded due to mismatches between the predicted genders and the clinically annotated genders.

*Metabolomics data preprocessing for replication cohorts*

NMR data preprocessing was conducted exactly like for KORA F4. For the YFS cohort, one sample was excluded due to a low detection rate (74.6%) for the metabolic measures and two samples were excluded because they were not in a state of fasting.

*Statistical analyses*

*Epigenome-wide discovery analysis*

The discovery EWAS were carried out using omicABEL (beta version, and with DatABEL v.0.9-6.) (7), a software package allowing rapid (epi-) genome-wide association analyses. As there were 10 MICE-imputed datasets, each EWAS was run 10 times and the coefficients and test statistics were combined using the method of (12) and the R package miceadds (13). A CpG site-metabolic measure pair was carried forward to further analysis if the p-value of the methylation coefficient was lower than a Bonferroni-corrected threshold of 0.05 / (226 x 468151) = 4.7e-10, for a family-wise type I error rate of 0.05.

*Sensitivity analysis and verification of model assumptions*

To investigate the validity of the regression models used in the discovery analysis we re-ran the models of the significant CpG-trait pairs, but firstly replacing the 0 values for the metabolic measures with NAs and using complete case analysis on all data, rather than any imputation methods. A model was considered unstable if the coefficient for the methylation changed by 50% from its original (discovery analysis) value or if the p-value increased by a factor of 100, resulting in the methylation coefficient no longer being statistically significantly different to 0 (p-value threshold 4.7e-10). The models failing these criteria were eliminated from future analysis. The genomic inflation factors for the 226 EWAS ranged from 0.9 to 1.3 across all metabolic measures (Additional File 19: Supplemental Table 14). Inflation factors were calculated using the R package MatrixEQTL (14). None of the resulting associated CpG sites have been reported to be cross-reactive probes (15, 16).

*Replication and meta-analysis*

The three replication studies detailed above ran linear models for the 274 pairs found to be robustly associated in the discovery analysis, adjusting for the same covariates, except where noted in the study-specific descriptions. A random effects meta-analysis was then performed using the replication results of the three participating studies, of the coefficients of the CpG sites from each of the 274 pairs examined, using the DerSimonian-Laird estimator (rma command of the R package metaphor, v1.9-8). As there were 274 pairs examined statistical significance was based on a Bonferroni-corrected threshold of p < 0.05 / 274 ≈ 1.8e-4.

We ran principal component analysis (PCA) on the (mean-imputed) metabolites in the discovery KORA F4 cohort and extracted the principal components (PCs) explaining a combined total variance of > 80%, in this case the first 8 PCs (Additional File 7: Supplemental Figure 2). For each of these PCs we ran an EWAS in the discovery KORA F4 cohort, with the PC as outcome and the methylation beta values as explanatory variable, adjusting for the same covariates as in the discovery analysis.  The threshold for statistical significance for association for a PC-CpG site pair was set at a Bonferroni-corrected threshold of p = 0.05 / (8 x 468151) = 1.34e-8.

*Multi-omics analyses*

*Genetic analysis*

The genetic data for KORA F4 were generated using the Affy Axiom chip and the pipeline described in (17), with prephasing in SHAPEIT v2 and imputation through IMPUTE v2.3.0 using 1000 Genome (phase 1 integrated haplotypes CEU, updated June 14, 2014) as the reference panel. Of the 1662 individuals of the discovery analysis, 1598 also had genetic/SNP data.

Using the KORA F4 data, we investigated genetic effects on the associations between methylation and metabolic measures replicated in the meta-analysis through conditional analysis. In more precise detail:

1. For each significant CpG site, we examined those SNPs within 1Mb. To determine if these SNPs and CpG sites were associated, for each pair we ran a linear regression additive model with methylation as outcome, and SNP dosage and all discovery analysis covariates as independent variables. Those pairs whose SNP coefficient achieved a p-value < 0.01 were carried forward to the next step.
2. For each SNP-metabolic measure pair for which both the SNP and the metabolic measure were associated with (at least) one common CpG site, we ran a linear regression additive model with metabolic measure value as outcome, and SNP dosage and all discovery analysis covariates as independent variables. Those pairs whose SNP coefficient achieved a p-value < 0.01 were carried forward to the next step.
3. For each SNP that showed association with each component of an associated CpG-metabolic measure pair, we re-ran the multiple imputation discovery analysis regression model, but including the SNP as a potential confounder. It was then determined if the CpG site lost statistical significance according to the discovery analysis threshold with the addition of any single SNP to the model.

*Gene expression analysis*

Gene expression data for KORA F4 were generated using the Illumina Human HT-12 v3 Expression BeadChip, with laboratory procedures, quality control and data pre-processing as described in (18), the final data being log-2 transformed and quantile normalized. Of the 1662 individuals from the discovery stage, 692 had gene expression data.

For each CpG site and each expression probe within 1Mb, we ran a mixed effects linear model (R package nlme v3.1-137) with log2 of the expression as outcome, and technically adjusted β-value, RNA integrity number, sample storage time and all covariates from the discovery analysis as fixed effects, and amplification plate as a random effect.

Statistical significance for an association between methylation of a given CpG site and a given expression probe was based on the p-value for the coefficient of the CpG site. A total of 480 CpG site-expression probe pairs were tested for association, giving a Bonferroni-corrected significance threshold of p < 0.05 / 480 ≈ 1.0e-4.

To extend the results to tissues other than whole blood, we extracted data from Array Express. Firstly, data on expression (ArrayExpress identifier E-TABM-1140, Illumina HumanHT-12 v3.0 Expression BeadChip) and methylation (E-MTAB-1866, Illumina Infinium HumanMethylation450 BeadChip) measured in subcutaneous fat from the same individuals in the TwinsUK study (N=626 overlap, all females, 241 twin pairs and 144 singletons) was extracted. The expression data had been previously quality controlled and processed by (19) and the methylation data by (20). As with the KORA F4 analysis, we looked at *cis* relationships (1 Mb) between expression probes and our CpG sites of interest, using the annotation files provided by the authors, performing correlation analysis using the R-package rmcorr v0.3.0 (function rmcorr) to take into account the correlation structure due to the twin pairs. We examined a total of 521 CpG-transcript pairs. Secondly, expression and methylation data from adult liver was extracted from Array Express reference E-GEOD-61279 (Illumina HumanHT-12 V4.0 Expression BeadChip), the data having been previously quality controlled and processed according to (21). Regression analyses were run for *cis* (as defined earlier) CpG-transcript pairs, with log-transformed expression as the outcome and methylation beta value as the independent variable, adjusting for sex and cancer case-control status (N=92 total, 42 cases). Statistical significance was based on a Bonferroni-corrected threshold of p < 0.05 / 271, as we examined 271 CpG-transcript pairs.

We looked up the significant CpG-transcript results in the BIOS QTL database (22, 23), from the *cis*-eQTM independent top effects (FDR < 0.05). CpG sites were matched by name, and transcripts were matched by annotated gene. Associations were considered replicated only if the directions of effect were consistent. The results from the BIOS database are based on *cis* being defined as the CpG site being found within a window of 250 kb around the transcription start site of the transcript. Further, *cis*-eQTLS and *cis*-mQTLs were firstly regressed out of the expression and methylation data, respectively, to ensure the associations were free of genetic influence. See Bonder, M. J., et al. (22) for full details.

*Associations with metabolic ratios from additional pathways*

Additional metabolic ratios, beyond those provided by the NMR platform, corresponding to amino acid, glucose, and lipid pathways were calculated in KORA F4 and assessed for association. Only metabolic measure-associated CpG sites that showed replication in the meta-analysis were included, and the same covariates from the epigenome-wide discovery analysis were used. Ratios implying enzymatic activity of glycolysis, gluconeogenesis, ketoacidosis and lactic acidosis pathways were included. Ratios linked to metabolic diseases were also incorporated, such as the branched-chain amino acid link to lipid metabolism as part of metabolic profiles related to diabetes (24); branched-chain amino acid relationship to glucose metabolism as it has been linked to insulin resistance, diabetes and obesity (24, 25); glycoprotein to lipid metabolism as a connection between low-grade inflammation and lipid metabolism (4); selected lipid ratios as measures for cardiovascular risk (26, 27); HDL content and size ratios and atherosclerosis (28, 29); polyunsaturated fatty acids as a possible link to cardiovascular and type 2 diabetes risk (30, 31); and cholesterol content in LDL and atherosclerosis and metabolic syndrome (32, 33). The tests for the associations of the 12 CpG sites with 30 transcripts of proteins directly involved in lipoprotein metabolism included transcripts of apolipoproteins such as five different types of ApoA, ApoB, three types of ApoC, ApoE, and ApoD apolipoproteins; enzymes such as lipoprotein lipase and hepatic lipase; transfer proteins such as cholesteryl ester transfer protein; and lipid transporters such as ATP Binding Cassette Subfamily A Member 1.

**References**

1. Zeilinger S, Kuhnel B, Klopp N, Baurecht H, Kleinschmidt A, Gieger C, et al. Tobacco smoking leads to extensive genome-wide changes in DNA methylation. PLoS One. 2013;8(5):e63812.

2. Lehne B, Drong AW, Loh M, Zhang W, Scott WR, Tan ST, et al. A coherent approach for analysis of the Illumina HumanMethylation450 BeadChip improves data quality and performance in epigenome-wide association studies. Genome Biol. 2015;16:37.

3. Aryee MJ, Jaffe AE, Corrada-Bravo H, Ladd-Acosta C, Feinberg AP, Hansen KD, et al. Minfi: a flexible and comprehensive Bioconductor package for the analysis of Infinium DNA methylation microarrays. Bioinformatics. 2014;30(10):1363-9.

4. Inouye M, Kettunen J, Soininen P, Silander K, Ripatti S, Kumpula LS, et al. Metabonomic, transcriptomic, and genomic variation of a population cohort. Mol Syst Biol. 2010;6:441.

5. Soininen P, Kangas AJ, Wurtz P, Suna T, Ala-Korpela M. Quantitative serum nuclear magnetic resonance metabolomics in cardiovascular epidemiology and genetics. Circ Cardiovasc Genet. 2015;8(1):192-206.

6. Houseman EA, Accomando WP, Koestler DC, Christensen BC, Marsit CJ, Nelson HH, et al. DNA methylation arrays as surrogate measures of cell mixture distribution. BMC Bioinformatics. 2012;13:86.

7. Fabregat-Traver D, Sharapov S, Hayward C, Rudan I, Campbell H, Aulchenko Y, et al. High-Performance Mixed Models Based Genome-Wide Association Analysis with omicABEL software. F1000Res. 2014;3:200.

8. van Buuren S, Groothuis-Oudshoorn K. mice: Multivariate Imputation by Chained Equations in R. 2011. 2011;45(3):67.

9. Bioconductor [Available from: <http://www.bioconductor.org/>.

10. Kananen L, Marttila S, Nevalainen T, Jylhava J, Mononen N, Kahonen M, et al. Aging-associated DNA methylation changes in middle-aged individuals: the Young Finns study. BMC Genomics. 2016;17:103.

11. Horvath S. DNA methylation age of human tissues and cell types. Genome Biol. 2013;14(10):R115.

12. Kim-Hung Li X-LM, T. E. Raghunathan and Donald B. Rubin. Significance levels from repeated p-values with multiply-imputed data. Institute of Statistical Science, Academia Sinica. 1991;1:65-92.

13. Robitzsch A GS. miceadds: Some Additional Multiple Imputation Functions, Especially for 'mice'. 2020;R package version 3.9-14.

14. Shabalin AA. Matrix eQTL: ultra fast eQTL analysis via large matrix operations. Bioinformatics. 2012;28(10):1353-8.

15. Chen YA, Lemire M, Choufani S, Butcher DT, Grafodatskaya D, Zanke BW, et al. Discovery of cross-reactive probes and polymorphic CpGs in the Illumina Infinium HumanMethylation450 microarray. Epigenetics. 2013;8(2):203-9.

16. Price ME, Cotton AM, Lam LL, Farre P, Emberly E, Brown CJ, et al. Additional annotation enhances potential for biologically-relevant analysis of the Illumina Infinium HumanMethylation450 BeadChip array. Epigenetics Chromatin. 2013;6(1):4.

17. Wahl A, van den Akker E, Klaric L, Stambuk J, Benedetti E, Plomp R, et al. Genome-Wide Association Study on Immunoglobulin G Glycosylation Patterns. Front Immunol. 2018;9:277.

18. Schurmann C, Heim K, Schillert A, Blankenberg S, Carstensen M, Dorr M, et al. Analyzing illumina gene expression microarray data from different tissues: methodological aspects of data analysis in the metaxpress consortium. PLoS One. 2012;7(12):e50938.

19. Grundberg E, Small KS, Hedman AK, Nica AC, Buil A, Keildson S, et al. Mapping cis- and trans-regulatory effects across multiple tissues in twins. Nat Genet. 2012;44(10):1084-9.

20. Grundberg E, Meduri E, Sandling JK, Hedman AK, Keildson S, Buil A, et al. Global analysis of DNA methylation variation in adipose tissue from twins reveals links to disease-associated variants in distal regulatory elements. Am J Hum Genet. 2013;93(5):876-90.

21. Bonder MJ, Kasela S, Kals M, Tamm R, Lokk K, Barragan I, et al. Genetic and epigenetic regulation of gene expression in fetal and adult human livers. BMC Genomics. 2014;15:860.

22. Bonder MJ, Luijk R, Zhernakova DV, Moed M, Deelen P, Vermaat M, et al. Disease variants alter transcription factor levels and methylation of their binding sites. Nat Genet. 2017;49(1):131-8.

23. Zhernakova DV, Deelen P, Vermaat M, van Iterson M, van Galen M, Arindrarto W, et al. Identification of context-dependent expression quantitative trait loci in whole blood. Nat Genet. 2017;49(1):139-45.

24. Wang TJ, Larson MG, Vasan RS, Cheng S, Rhee EP, McCabe E, et al. Metabolite profiles and the risk of developing diabetes. Nat Med. 2011;17(4):448-53.

25. Newgard CB, An J, Bain JR, Muehlbauer MJ, Stevens RD, Lien LF, et al. A branched-chain amino acid-related metabolic signature that differentiates obese and lean humans and contributes to insulin resistance. Cell Metab. 2009;9(4):311-26.

26. Piperi C, Kalofoutis C, Papaevaggeliou D, Papapanagiotou A, Lekakis J, Kalofoutis A. The significance of serum HDL phospholipid levels in angiographically defined coronary artery disease. Clin Biochem. 2004;37(5):377-81.

27. Niemi J, Makinen VP, Heikkonen J, Tenkanen L, Hiltunen Y, Hannuksela ML, et al. Estimation of VLDL, IDL, LDL, HDL2, apoA-I, and apoB from the Friedewald inputs--apoB and IDL, but not LDL, are associated with mortality in type 1 diabetes. Ann Med. 2009;41(6):451-61.

28. Bagheri B, Alikhani A, Mokhtari H, Rasouli M. The Ratio of Unesterified/esterified Cholesterol is the Major Determinant of Atherogenicity of Lipoprotein Fractions. Med Arch. 2018;72(2):103-7.

29. Huuskonen J, Olkkonen VM, Jauhiainen M, Ehnholm C. The impact of phospholipid transfer protein (PLTP) on HDL metabolism. Atherosclerosis. 2001;155(2):269-81.

30. Wang L, Folsom AR, Eckfeldt JH. Plasma fatty acid composition and incidence of coronary heart disease in middle aged adults: the Atherosclerosis Risk in Communities (ARIC) Study. Nutr Metab Cardiovasc Dis. 2003;13(5):256-66.

31. Rhee EP, Cheng S, Larson MG, Walford GA, Lewis GD, McCabe E, et al. Lipid profiling identifies a triacylglycerol signature of insulin resistance and improves diabetes prediction in humans. J Clin Invest. 2011;121(4):1402-11.

32. Chait A, Ginsberg HN, Vaisar T, Heinecke JW, Goldberg IJ, Bornfeldt KE. Remnants of the Triglyceride-Rich Lipoproteins, Diabetes, and Cardiovascular Disease. Diabetes. 2020;69(4):508-16.

33. Srisawasdi P, Vanavanan S, Rochanawutanon M, Kruthkul K, Kotani K, Kroll MH. Small-dense LDL/large-buoyant LDL ratio associates with the metabolic syndrome. Clin Biochem. 2015;48(7-8):495-502.
