# Supplementary figures and images for: DNA methylation and lipid metabolism: an EWAS of 226 metabolic measures
Source: Clin Epigenetics. 2021 Jan 7;13:7. doi: 10.1186/s13148-020-00957-8 (PMC7789600; doi:10.1186/s13148-020-00957-8)

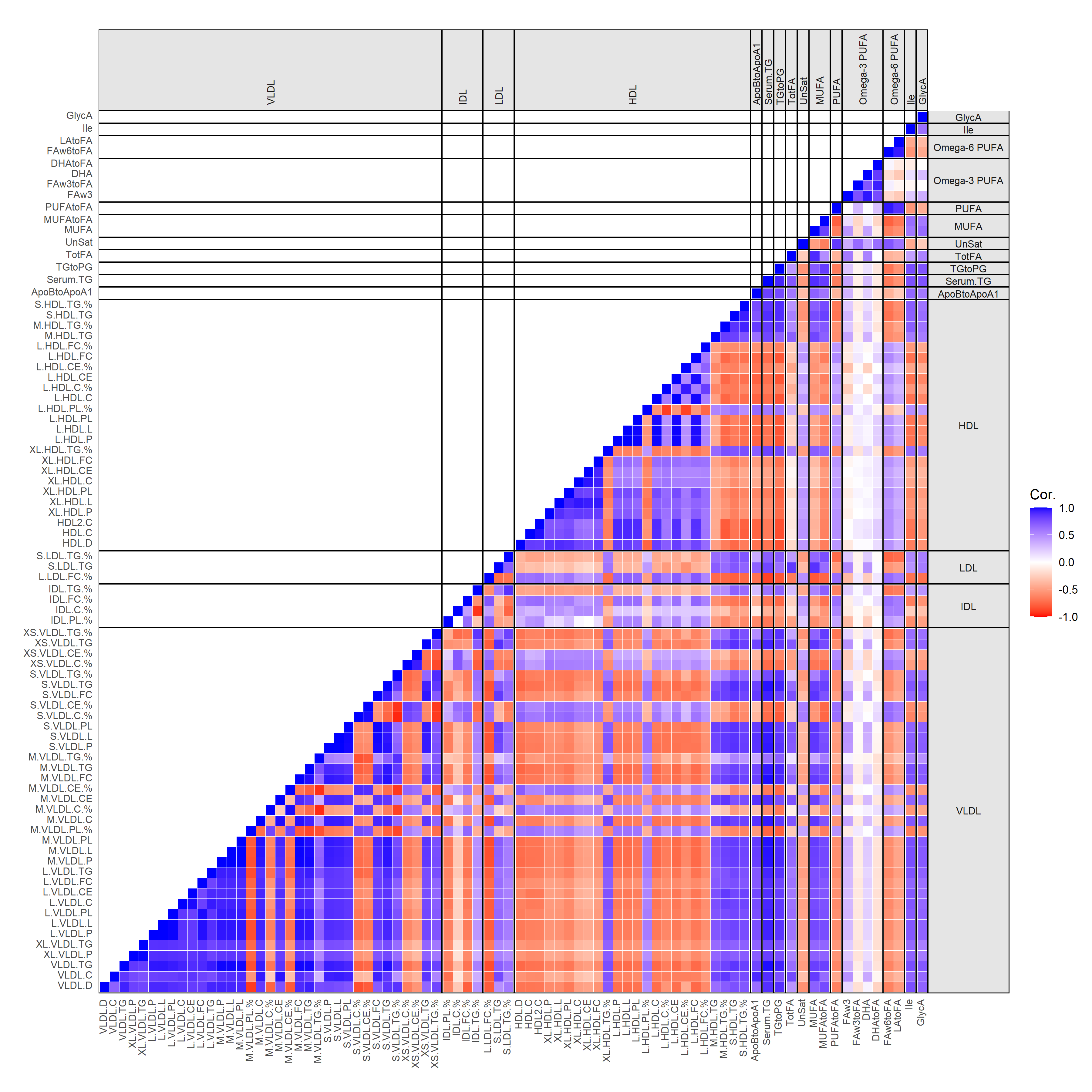

Supplement: Supplementary file 6 — Additional file 6: Figure S1. Pearson correlations between metabolic measures associated with DNA methylation, KORA F4 data. Correlations among DNA methylation-associated metabolic measures are shown. All metabolic measures found associated with methylation in the discovery cohort are included. [file 13148_2020_957_MOESM6_ESM.png]

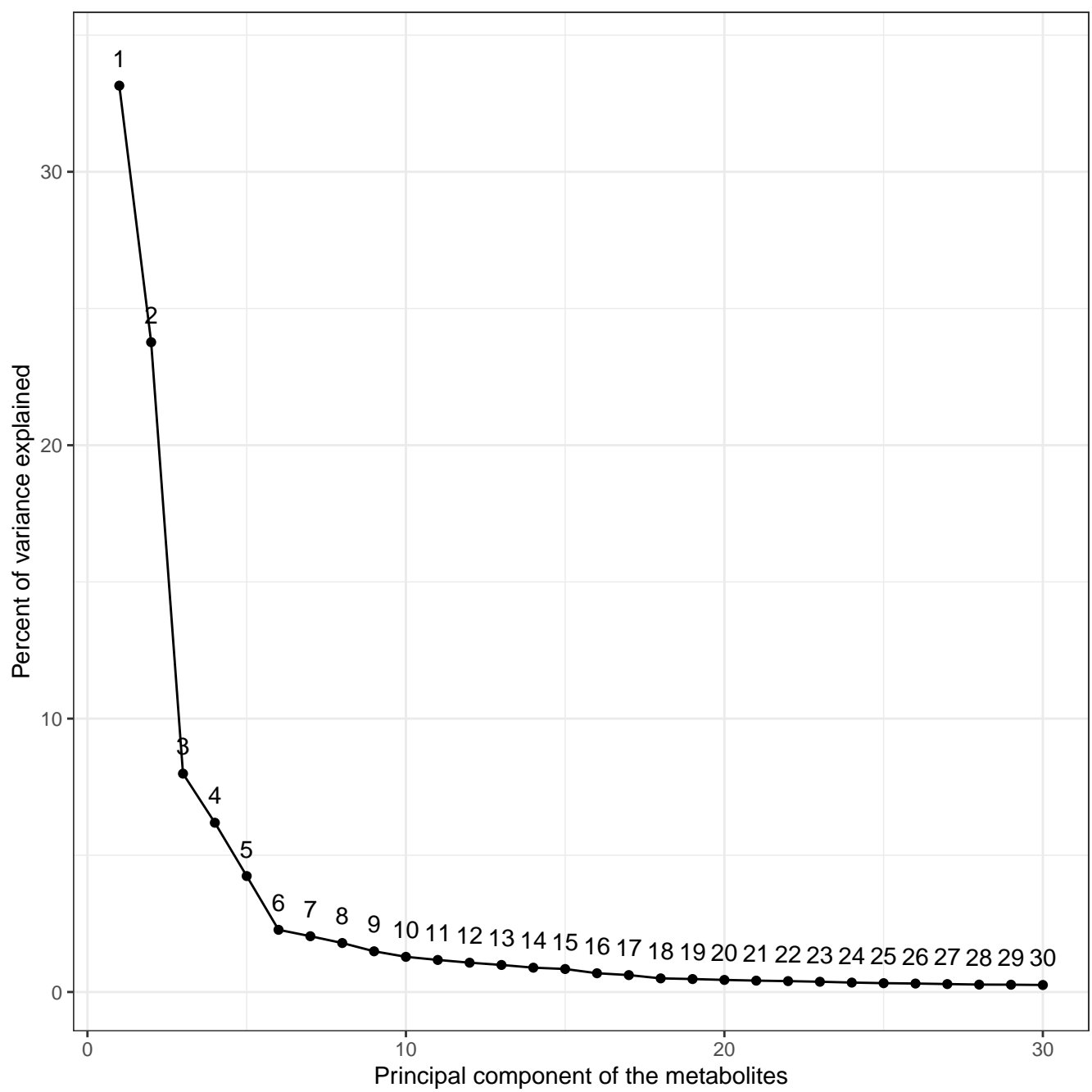

Supplement: Supplementary file 7 — Additional file 7: Figure S2. Explained variance of the first 30 principal components of the metabolite measure principal component analysis, performed in the discovery cohort KORA F4. [file 13148_2020_957_MOESM7_ESM.pdf]
