# Supplementary material for: DNA methylation and lipid metabolism: an EWAS of 226 metabolic measures
Source: Clin Epigenetics. 2021 Jan 7;13:7. doi: 10.1186/s13148-020-00957-8 (PMC7789600; doi:10.1186/s13148-020-00957-8)
Supplement: Supplementary file 8 — Additional file 8: Figure S3. Presented are the first 8 principal components (PCs) of the metabolite data in KORA F4 (after scaling of the individual metabolites) coloured according to sex, intake of lipid-lowering drugs, and smoking habits. “Expl. Var” is the variance explained by the given PC. Some clustering is observed for sex within the first 2 PCs, but no other obvious clusters emerge for any of the other phenotypes or PCs. [file 13148_2020_957_MOESM8_ESM.pdf]

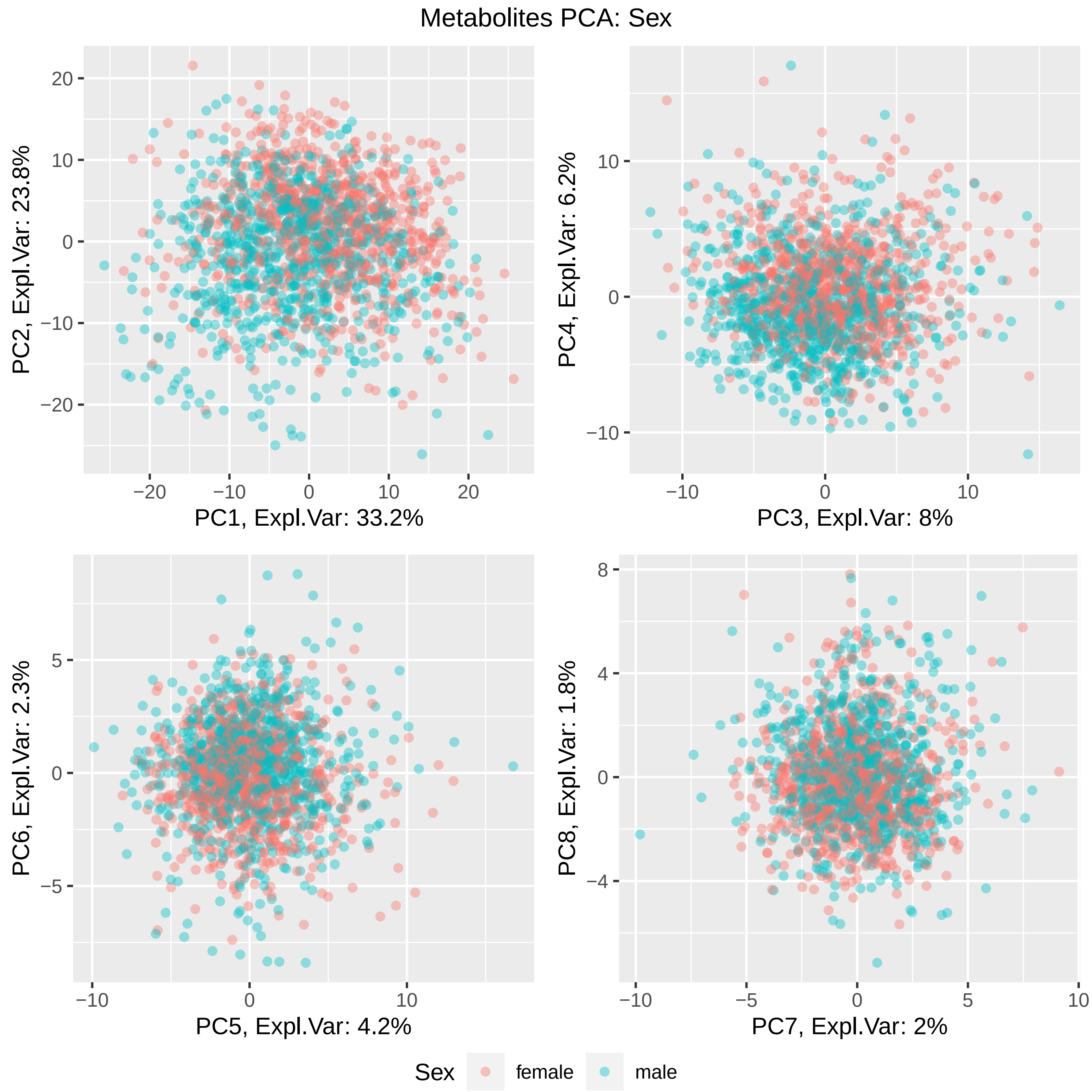

Metabolites PCA: Sex
PC1, Expl.Var: 33.2%
PC2, Expl.Var: 23.8%
PC3, Expl.Var: 8%
PC4, Expl.Var: 6.2%
PC5, Expl.Var: 4.2%
PC6, Expl.Var: 2.3%
PC7, Expl.Var: 2%
PC8, Expl.Var: 1.8%
Sex
female
male

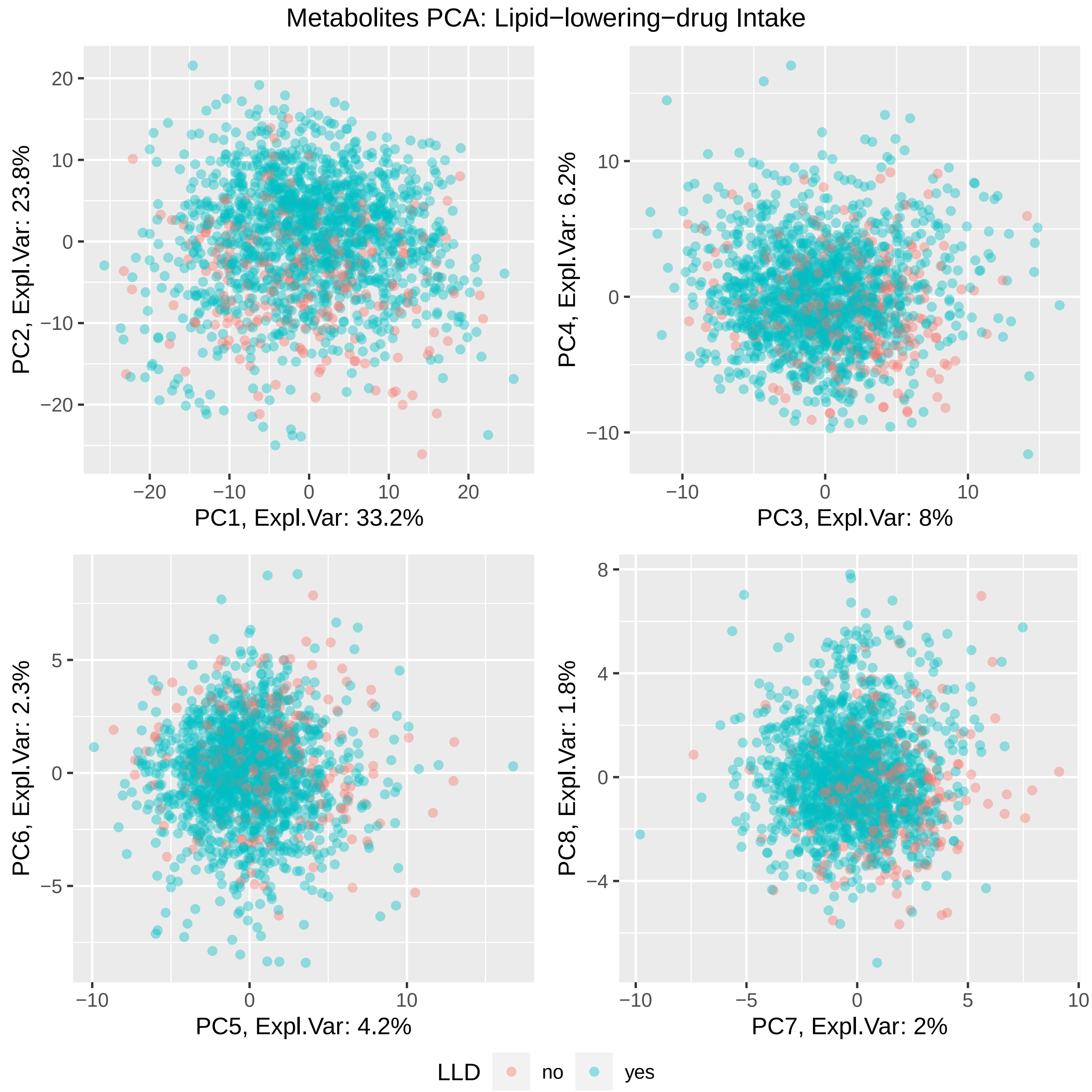

Metabolites PCA: Lipid−lowering−drug Intake
PC1, Expl.Var: 33.2%
PC2, Expl.Var: 23.8%
PC3, Expl.Var: 8%
PC4, Expl.Var: 6.2%
PC5, Expl.Var: 4.2%
PC6, Expl.Var: 2.3%
PC7, Expl.Var: 2%
PC8, Expl.Var: 1.8%
LLD
no
yes

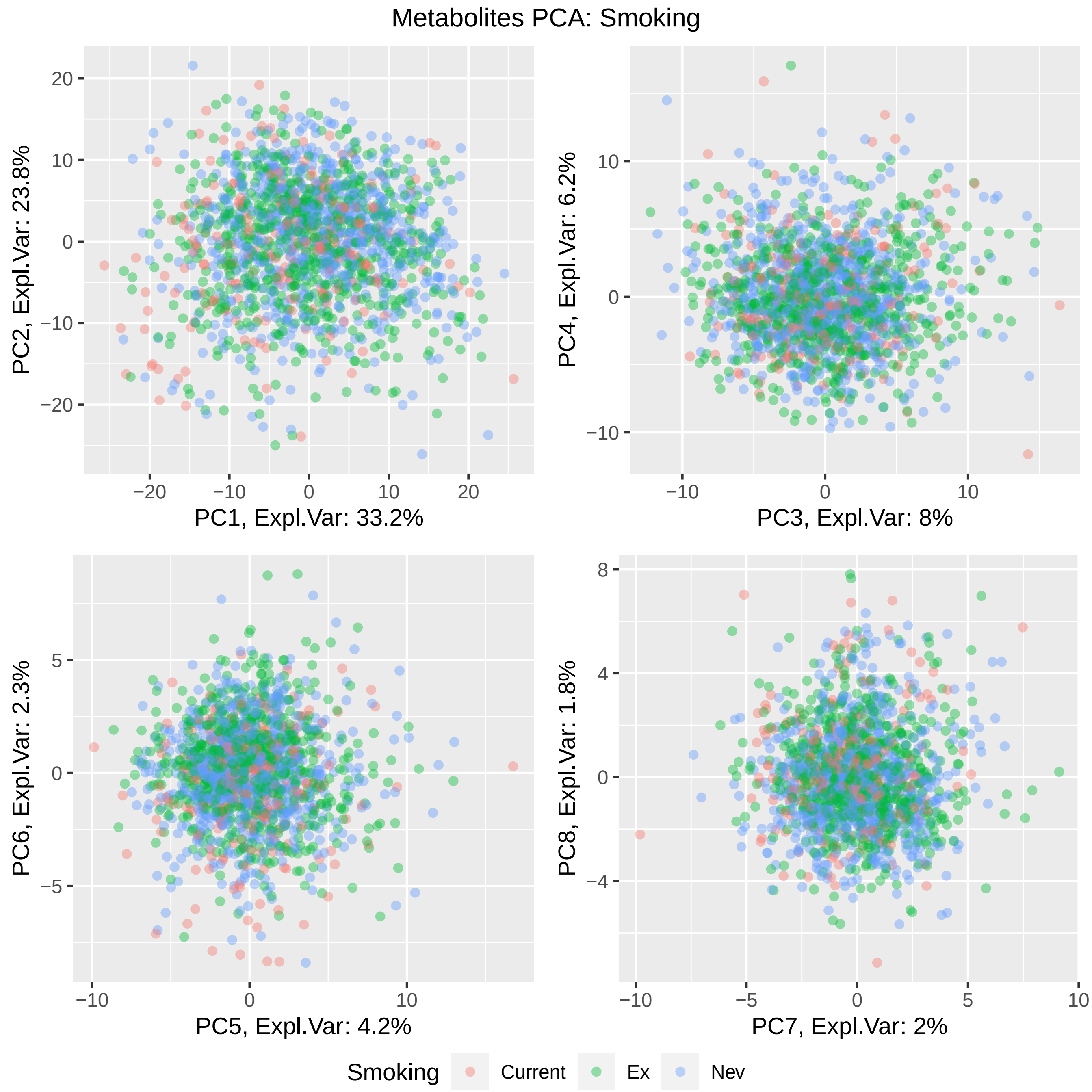

Metabolites PCA: Smoking
PC1, Expl.Var: 33.2%
PC2, Expl.Var: 23.8%
PC3, Expl.Var: 8%
PC4, Expl.Var: 6.2%
PC5, Expl.Var: 4.2%
PC6, Expl.Var: 2.3%
PC7, Expl.Var: 2%
PC8, Expl.Var: 1.8%
Smoking
Current
Ex
Nev
