# Supplementary material for: DNA methylation and lipid metabolism: an EWAS of 226 metabolic measures
Source: Clin Epigenetics. 2021 Jan 7;13:7. doi: 10.1186/s13148-020-00957-8 (PMC7789600; doi:10.1186/s13148-020-00957-8)

Supplemental figure 4: ROC comparison

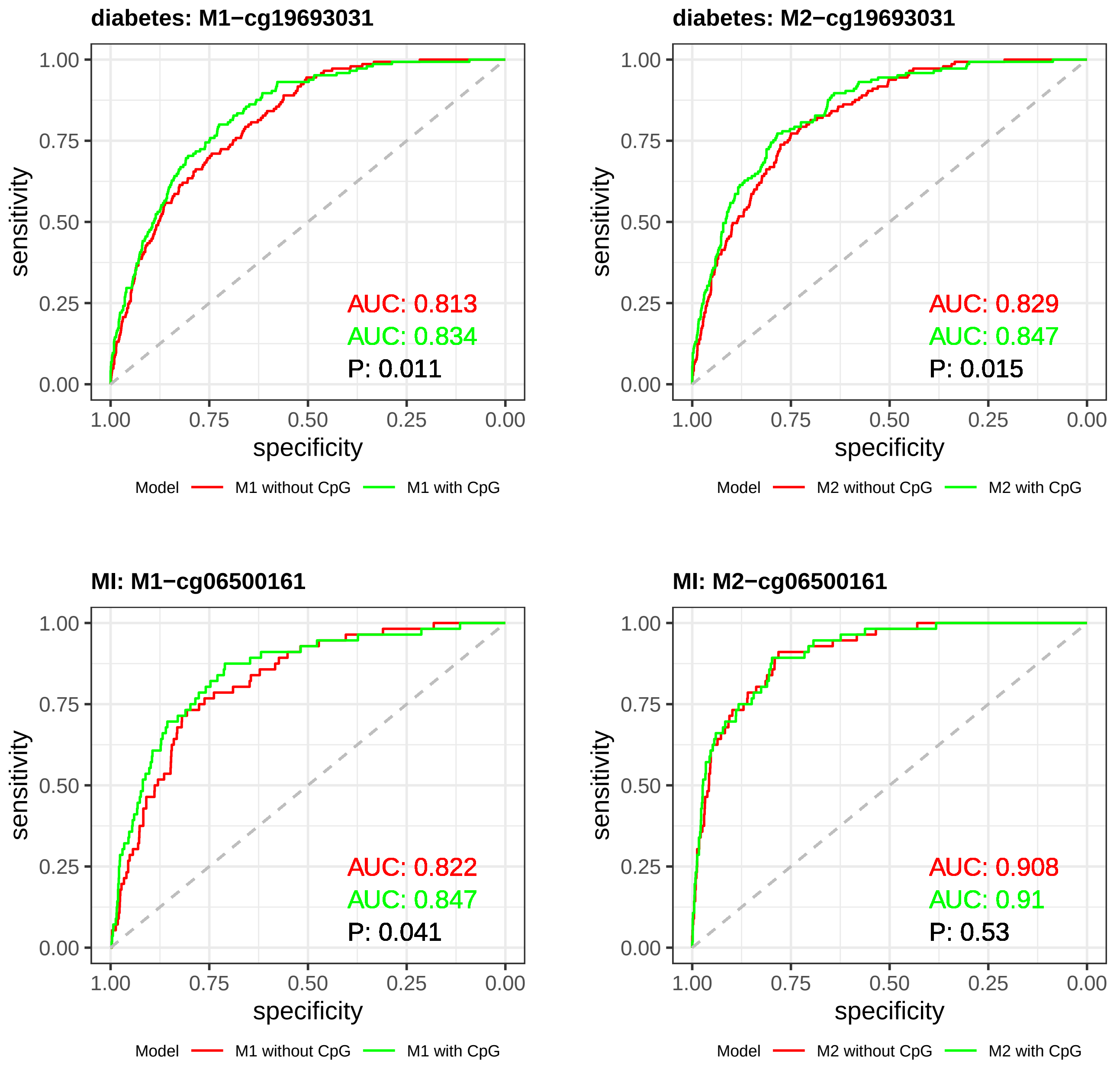

Supplemental figure 4: ROC comparison

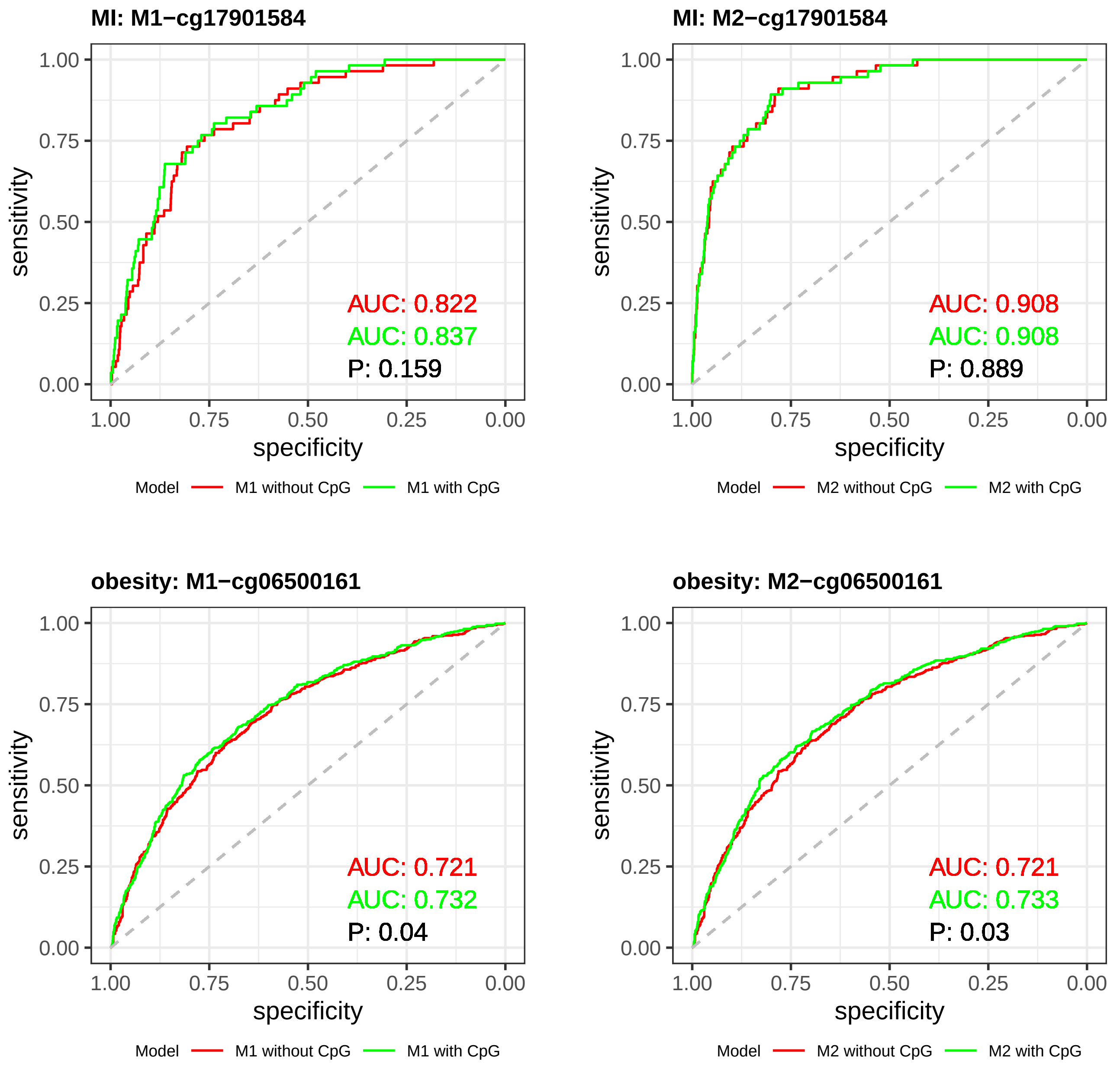

Supplemental figure 4: ROC comparison

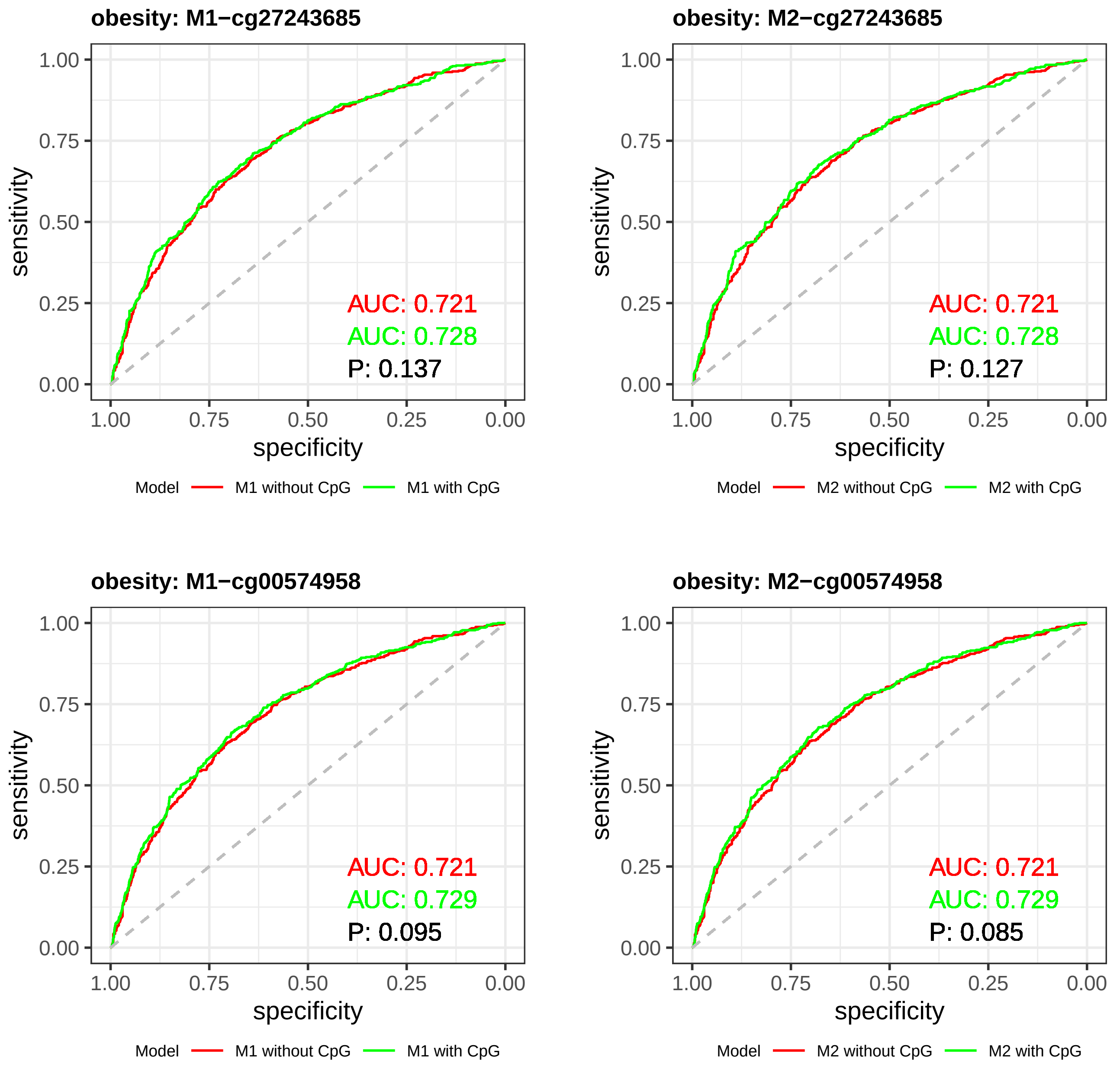

Supplemental figure 4: ROC comparison

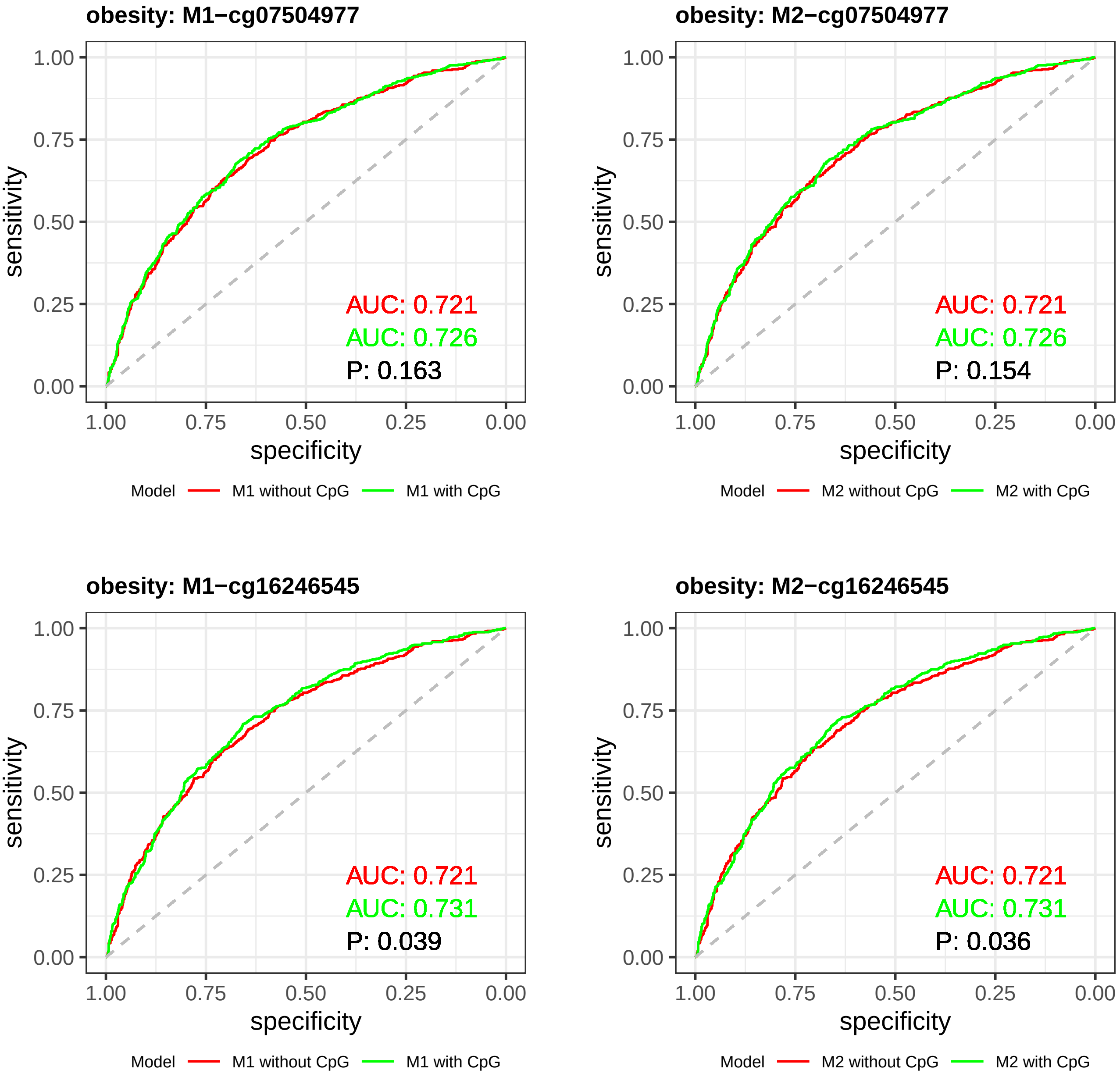

Supplemental figure 4: ROC comparison

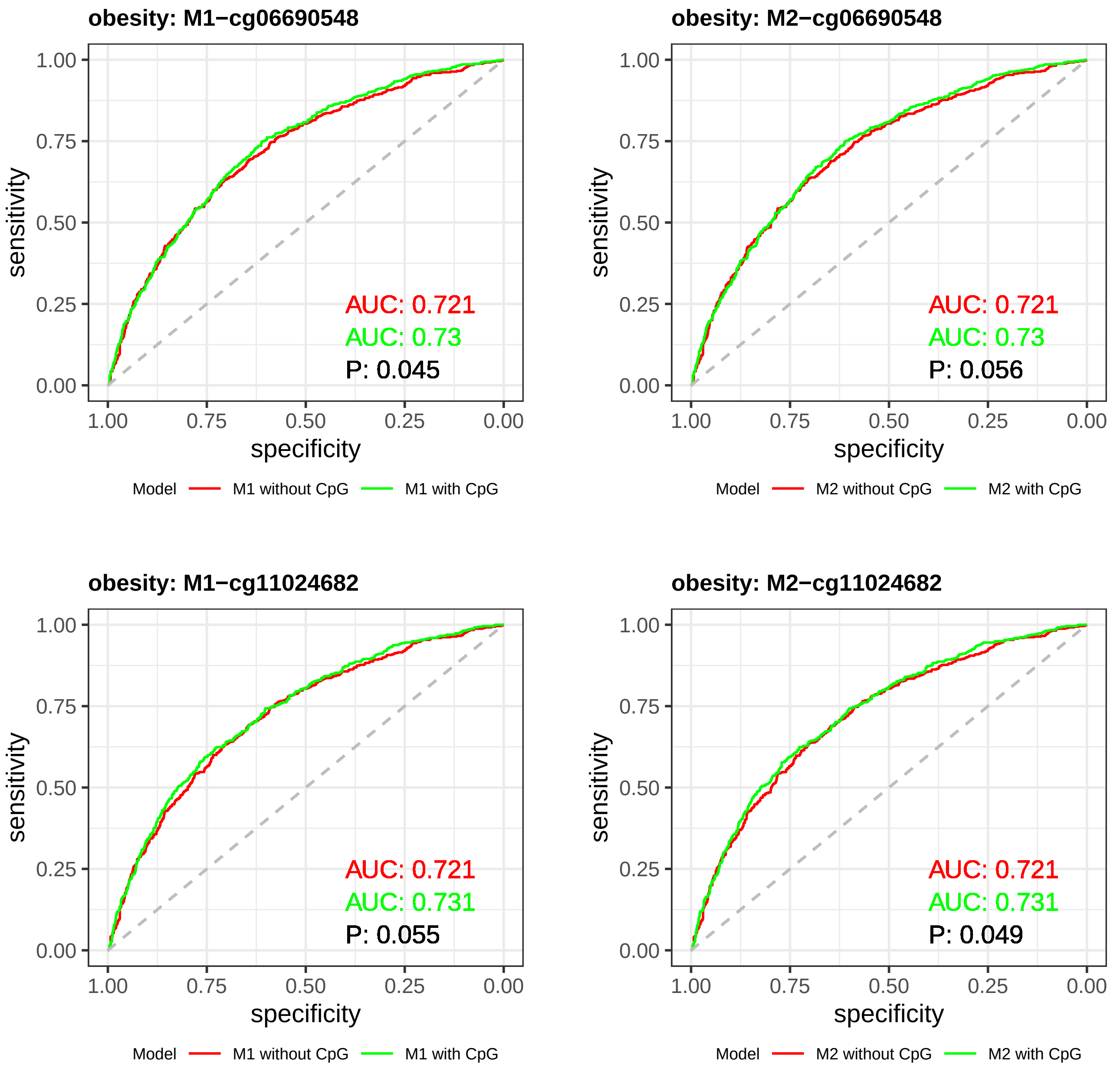

Supplement: Supplementary file 18 — Additional file 18: Figure S4. ROC curves for significant CpG-outcome associations: Presented are the receiver operating characteristic (ROC) curves for each outcome-CpG pair for which there exists a statistically significant association for either M1 or M2 (Table 3). The red line is the ROC curve for the model without the CpG, and the green line is the model with the CpG. Presented are also the areas under the curve (AUC) for the respective ROCs, and a p value for the null hypothesis that the addition of the CpG to the model has no effect on the predictive performance of the model. The p value was determined using the R package pROC [87], command roc.test, method “bootstrap”. The analysis was run in the KORA F4 dataset, and, to ensure comparability of the results, the ROC curves were generated using individuals with no missing values in any of the outcomes or covariates, and the methylation data were mean imputed. [file 13148_2020_957_MOESM18_ESM.pdf]
